# Exploring the heterogeneity of hepatic and pancreatic fat deposition in obesity: implications for metabolic health

**DOI:** 10.3389/fendo.2024.1447750

**Published:** 2024-10-08

**Authors:** Ming Deng, Zhen Li, Shangyu Chen, Huawei Wang, Li Sun, Jun Tang, Liman Luo, Xiaoxiao Zhang, Haibo Xu, Zhe Dai

**Affiliations:** ^1^ Department of Radiology, Zhongnan Hospital of Wuhan University, Wuhan University, Wuhan, China; ^2^ Hubei Provincial Engineering Research Center of Multimodal Medical Imaging Technology and Clinical Application, Wuhan, China; ^3^ Wuhan Clinical Research and Development Center of Brain Resuscitation and Functional Imaging, Wuhan, China; ^4^ Department of Hepatobiliary and Pancreatic Surgery, Zhongnan Hospital of Wuhan University, Wuhan University, Wuhan, China; ^5^ Department of Endocrinology, Zhongnan Hospital of Wuhan University, Wuhan University, Wuhan, China; ^6^ Department of Endocrinology and Metabolism, Guangxi Academy of Medical Sciences and the People’s Hospital of Guangxi Zhuang Autonomous Region, Guangxi, Nanning, China; ^7^ Department of MSC Clinical & Technical Solutions, Philips Healthcare, Beijing, China; ^8^ Department of Clinical Nutrition, Zhongnan Hospital of Wuhan University, Wuhan University, Wuhan, China

**Keywords:** obesity, non-alcoholic fatty liver disease, non-alcoholic fatty pancreas disease, insulin resistance, fat deposition heterogeneity

## Abstract

**Objective:**

This retrospective observational study investigates the heterogeneity of hepatic and pancreatic fat deposition and its implications for metabolic health in individuals with obesity.

**Methods:**

A total of 706 patients with obesity underwent an MRI to quantify liver and pancreatic fat. Patients were classified into four groups based on fat deposition: no fat (None), fatty pancreas only (NAFPD), fatty liver only (NAFLD), and both conditions (NAFLD+NAFPD). Biochemical profiles, insulin resistance (Homeostatic Model Assessment for Insulin Resistance, HOMA-IR), and β-cell function were analyzed. A series of multiple linear regressions were used to investigate the independent effects of characteristics on glucose, insulin, and C-peptide at 0h. Another multiple linear regression was performed to evaluate the effects of basic characteristics on average liver fat, mean pancreatic fat, and visceral fat.

**Results:**

The majority (76.63%) exhibited both NAFLD and NAFPD, highlighting the heterogeneity of fat deposition among individuals with obesity. Groups with fatty liver displayed significantly higher fasting glucose, insulin, C-peptide, and HOMA-IR levels than those without fatty liver (P < 0.01). Fatty pancreas alone did not significantly influence these metabolic parameters (P > 0.05). This underscores the greater metabolic impact of hepatic fat compared to pancreatic fat.

**Conclusions:**

The study confirms the complex heterogeneity of fat deposition in obesity, with the fatty liver being a more influential factor in metabolic disturbances than the fatty pancreas. The prevalent co-occurrence of NAFLD and NAFPD in this population underscores the need for targeted management strategies focusing on hepatic fat reduction to mitigate metabolic risk.

## Introduction

Obesity, a global health concern, is characterized not just by excessive body weight but also by abnormal fat distribution, which exerts profound implications for metabolic health. Central to this discussion is the heterogeneity of visceral fat deposition, particularly in the liver and pancreas, and its diverse impacts on metabolic health.

The distribution of adipose tissue in obesity profoundly influences metabolic and cardiovascular risk. Visceral fat, unlike subcutaneous fat, is strongly associated with metabolic disorders, including insulin resistance, type 2 diabetes, dyslipidemia, and cardiovascular disease (1–3). The pathophysiological mechanisms involve the secretion of pro-inflammatory cytokines and adipokines from visceral fat, thus disrupting insulin signaling and lipid metabolism (4–7).

Fatty liver disease, or hepatic steatosis, is a frequent manifestation of ectopic fat deposition in obesity. Non-alcoholic fatty liver disease (NAFLD), in particular, has arisen as a major concern due to its association with insulin resistance and its progression to more severe liver conditions, such as non-alcoholic steatohepatitis (NASH), fibrosis, and cirrhosis (8). The liver fat content, beyond exerting local effects, exerts widespread influence on systemic metabolism, affecting lipid profiles and glucose homeostasis (9, 10).

Parallel to the liver, the pancreas is another critical organ affected by ectopic fat deposition. Fatty pancreas, or non-alcoholic fatty pancreas disease (NAFPD), though less studied than NAFLD, has gained attention for its potential impact on β-cell function and insulin secretion (11–13). The interplay between fatty liver and pancreas in the context of obesity has been the subject of recent research, which has revealed a complex picture of metabolic health in individuals with obesity (14–16).

Despite the recognition of these patterns, the heterogeneity in fat deposition and its differential impact on metabolic health remains underexplored. Several studies have highlighted the variability in the distribution of visceral fat and its distinct metabolic consequences (10, 17). However, there is a need for a more nuanced understanding of how these variations in fat deposition, particularly in the liver and pancreas, manifest in terms of metabolic health.

The current study aims to bridge this gap by categorizing individuals with obesity based on the presence of fatty liver and pancreas, exploring the metabolic implications of these categories. This approach is novel, providing valuable insights into the heterogeneity of fat deposition in obesity and its broader metabolic implications.

In conclusion, understanding the heterogeneity in visceral fat deposition, particularly in the liver and pancreas, is critical to comprehending the full spectrum of metabolic health in obesity. The findings from this study could potentially pave the way for more personalized and effective strategies in managing and treating obesity and its related metabolic disorders.

## Material and methods

### Study population

In this retrospective analysis, we enrolled a cohort of 706 subjects who were admitted to Zhongnan Hospital of Wuhan University from August 2020 through January 2023. Each participant was assessed using visceral magnetic resonance imaging (MRI) to quantify adipose tissue and underwent a thorough set of anthropometric and biochemical assessments throughout their hospitalization. Eligible participants were adults aged 18 to 60 years with a body mass index (BMI) of 28 kg/m^2 or greater. Inclusion criteria demanded completion of visceral fat MRI quantification and documentation of anthropometric measures, including height, weight, waist circumference, and hip circumference. Participants who had undergone an oral glucose tolerance test (OGTT), an insulin release test (IRT), and a C peptide release test (CRT) were also included. Exclusion criteria encompassed individuals with liver injury induced by medications, autoimmune diseases, viral or cholestatic liver diseases, cirrhosis, secondary causes of obesity, past or present pancreatic cancer or pancreatitis, acute severe infections, recent cardiovascular or cerebrovascular events, pregnancy or breastfeeding, significant chronic ailments such as cancer or cardiac or renal failure, daily alcohol intake exceeding 20 grams, and those receiving glucocorticoid or anti-obesity medications. This study was conducted under the auspices of the Zhongnan Hospital of Wuhan University’s Ethics Committee, which approved (protocol number 2021019). All participant data were meticulously gathered and scrutinized following the highest ethical standards.

### MRI protocol and image analysis

Eligible patients in this study underwent abdominal MRI scans using a 3.0 T Ingenia CX system (Philips Healthcare, Best, The Netherlands), operated by experienced MRI technicians. The mDIXON Quant pulse sequence from Philips Healthcare was utilized for quantitative imaging, facilitating the assessment of liver and pancreatic fat content. Key parameters of the pulse sequence included the employment of multipoint DIXON techniques with a low flip angle of 3-5° to reduce T1 bias, the acquisition of six echoes for T2* correction, and a multipeak fat model application. The field of view was established at 500mm by 450mm, with a voxel size of 2.5mm by 2.5mm, a repetition time of 10ms, an echo time of 5ms, and a slice thickness set at 6mm.

Image acquisition stability was verified using a test group of ten volunteers. Axial images, capturing abdominal fat quantity from the diaphragmatic dome to the sacrum, were obtained in a single breath hold. Post-acquisition, Philips software automatically generated maps for water, fat, fat fraction (FF), and T2*map. Liver and pancreatic FF measurements for each participant were independently assessed by two readers, boasting 8 and 14 years of experience in abdominal-pelvis imaging, respectively. Regions of interest (ROIs) were meticulously and repeatedly outlined on abdominal FF maps for this purpose. Hepatic FF evaluation involved the manual placement of ROIs across eight liver segments, carefully avoiding the biliary tree and large vein vessels (see **Supplementary Figure S1** for details). Pancreatic FF was similarly measured, with ROIs placed within the head, body, and tail of the pancreas, excluding the splenic veins. Both reviewers were blinded to each other’s fat measurement results. The final data utilized for analysis represented the average of measurements obtained by both reviewers.

### Patient grouping for MRI-based fatty liver and pancreas analysis

Patients were stratified in this study following MRI criteria for fat quantification in the liver and pancreas (**Supplementary Table S1**), employing an FF threshold of greater than 5% to delineate fatty liver and pancreatitis (16). The cohort was categorized into four distinct groups based on the occurrence of fatty infiltrates in either organ in the population with obesity. Group 1, labeled ‘None’, encapsulated individuals devoid of fat accumulation in both the liver and pancreas. Group 2, designated ‘NAFPD’, comprised patients with fatty pancreas absent of fatty liver manifestations. Conversely, Group 3, known as ‘NAFLD’, encompassed patients with evidence of fatty liver but no fatty pancreas. The patients with coexisting fat in both the liver and pancreas were classified under Group 4, referred to as ‘NAFLD+NAFPD.’ The subgroup analysis delved into the degree of fat accumulation within the liver and pancreas, partitioning the participants into three grades of severity hinged upon FF metrics: mild (5%≦FF<15%), moderate (15%≦FF<25%), and severe (FF≥25%). These gradations were uniformly applied to the NAFLD and the NAFPD subgroups.

### Biochemical measurements

Biochemical profiling was procured by analyzing venous blood samples from fasting participants for 8 hours or longer. The spectrum of these indices spanned fasting blood glucose (BG), uric acid (UA), and an expansive lipid panel inclusive of total cholesterol (TC), triglycerides (TG), low-density lipoprotein (LDL), small and dense LDL-cholesterol (sdLDL-c), high-density lipoprotein (HDL), and lipoprotein a (Lpa). In addition, liver function was interrogated through markers such as alanine aminotransferase (ALT) and aspartate aminotransferase (AST), while renal health was gauged via blood urea nitrogen (BUN) and creatinine (CREA). These measurements were effectuated with Beckman AU5800 automated chemistry analyzers. Insulin and C-peptide concentrations were also ascertained using a proprietary chemiluminescence assay kit tailored for a specialized hormone autoanalyzer.

### Calculation method of BMI, insulin sensitivity, and β cell function

The calculation methods employed in this study for assessing various health metrics are as follows: BMI was determined by dividing an individual’s weight in kilograms by the square of their height in meters. To evaluate insulin sensitivity, the HOMA-IR (HOMA-IR = (fasting insulin (mU/L) × fasting glucose (mmol/L))/22.5) was utilized. Assessment of beta cell function was carried out using the Homeostasis Model Assessment of Beta Cell Function (HOMA-β, HOMA-β: 20 × fasting plasma insulin (mU/L)/(fasting plasma glucose (mmol/L) - 3.5)) and the Oral Glucose Insulin Sensitivity Index (DIO).

### Statistical analysis

The eligible participants were categorized into four groups: G1 (none), G2 (NAFPD), G3 (NAFLD), and G4 (NAFLD+NAFPD). We conducted statistical descriptions and comparisons of characteristics across these groups. Categorical variables were described using frequencies and percentages, and the Pearson χ2 test assessed statistical significance. For continuous variables, we reported the median (Q1, Q3) for description in cases of abnormality, and differences among groups were tested using the Kruskal-Wallis H test. Additionally, Spearman rank-order correlation was employed to assess the relationship between continuous characteristics and grade level across the four groups (G1, G2, G3, G4). The distributions of glucose, insulin, and C-peptide among the four groups at various time points (0h, 0.5h, 1h, 2h, and 3h) were visualized using boxplots. At each time point, differences among the groups were assessed using the Kruskal-Wallis H test, followed by *post-hoc* pairwise comparisons with Bonferroni correction. Concurrently, Spearman rank-order correlation was used to explore potential linear correlations. We conducted a series of multiple linear regressions to further investigate the independent effects of characteristics on glucose, insulin, and C-peptide at 0h. These models included basic characteristics as independent variables, with the group variable of interest (using “G1: none” as the reference) also incorporated. Another multiple linear regression was performed to evaluate the effects of basic characteristics on average liver fat, incorporating mean pancreatic fat and visceral fat area as additional independent variables. In all regression models, coefficients and corresponding 95% confidence interval (CI) were reported. Multi-collinearity was assessed using the variance inflation factor (VIF). A VIF greater than 10 for any included independent variable indicated potential serious multi-collinearity, which required corrective measures. Data analysis and visualization were conducted using R version 4.2.2 software (The R Foundation for Statistical Computing, Vienna, Austria; www.r-project.org) with the ggplot2 package. A two-sided p-value of less than 0.05 was considered to be statistically significant.

## Results

### Baseline characteristics analysis of four groups

**Figure 1:**
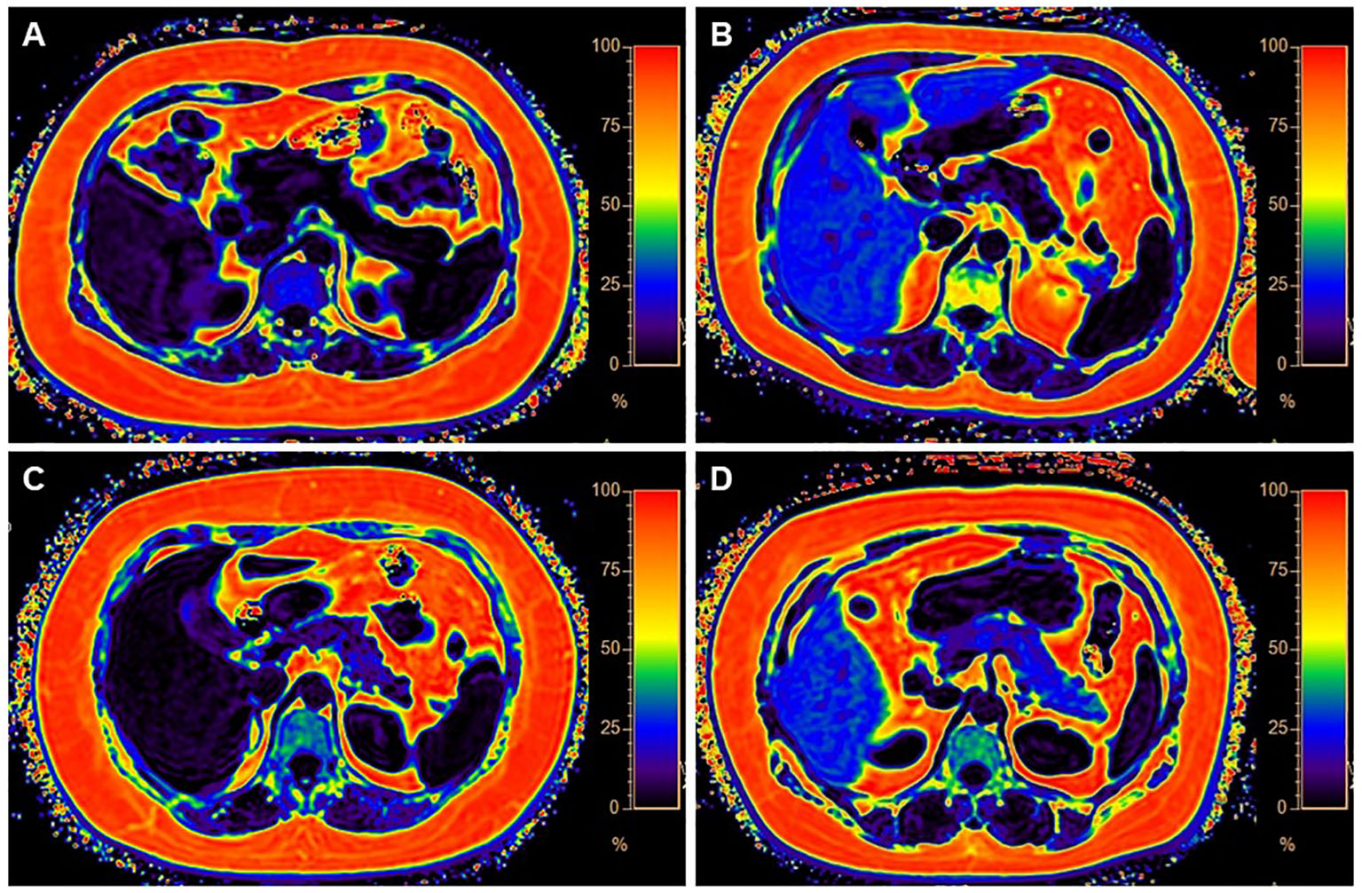
Four categories of MR images illustrate different levels of fat deposition in obesity. **(A)** Represents the first category, showing no fat deposition in either the liver or pancreas. The average fat fraction (FF) of the liver and pancreas were 4.37% and 1%, respectively. **(B)** Represents the second category, with high-fat deposition in the liver and minimal fat deposition in the pancreas. The average FF of the liver and pancreas were 28% and 4%, respectively. **(C)** Illustrates the third category, with less fat deposition in the liver but higher fat deposition in the pancreas. The average FF of the liver and pancreas were 3% and 13.1%, respectively. **(D)** Demonstrates the fourth category, characterized by fatty deposits in both the liver and pancreas. The average FF of the liver and pancreas were 31% and 22.6%, respectively.

The subjects, who were patients diagnosed with obesity, were stratified into four distinct groups based on the presence or absence of fatty liver and fatty pancreas. Group 1, designated as ‘None’, comprised 39 subjects (5.52% of the total), exhibiting neither fatty liver nor fatty pancreas. Group 2, labeled ‘NAFPD’, consisted of 16 subjects (2.27%), characterized by the presence of fatty pancreas but the absence of fatty liver. Group 3, termed ‘NAFLD’, included 110 subjects (15.58%), who exhibited fatty liver without fatty pancreas. Finally, Group 4, named ‘NAFLD+NAFPD’, was the largest, including 541 subjects (76.63%), characterized by the presence of both fatty liver and pancreas (**Table 1**). This distribution indicates that while a majority of patients with obesity concurrently exhibit both conditions, a significant proportion (approximately one-quarter) do not, underscoring the heterogeneity in ectopic fat deposition among patients with obesity. **Figure 1** provides a detailed illustration of the varying distributions of fatty liver and pancreas among the four groups. Compared to the other groups, Group 4 exhibits a higher proportion of males. Group 1 has a lower BMI relative to the other groups. Furthermore, Groups 1 and 2 display lower levels of TG, FFA, ALT, and uric acid compared to Groups 3 and 4, indicating that the combination of fatty liver in people with obesity is associated with more pronounced metabolic abnormalities (**Table 1**).

**Table 1:** Characteristics of study population and group comparisons.

Characteristics	Overall (N=706)	G1: None (n=39)	G2: NAFPD (n=16)	G3: NAFLD (n=110)	G4: NAFLD+NAFPD (n=541)	Z/χ^2^	*P*	r (*P*)
Gender, n (%)						13.19	0.004	–
Male	127 (18.01%)	3 (7.89%)	2 (12.50%)	9 (8.18%)	113 (20.89%)			
Female	578 (81.99%)	35 (92.11%)	14 (87.50%)	101 (91.82%)	428 (79.11%)			
Age, year	33 (28, 38)	32 (24, 39)	32 (30, 34)	31 (28, 37)	33 (28, 38)	2.101	0.552	0.05 (0.16)
BMI, kg/m^2^	35.19 (32.51, 39.10)	32.74 (30.06, 37.37)	34.43 (33.68, 36.81)	34.62 (32.66, 38.39)	35.65 (32.77, 39.45)	8.964	**0.030**	**0.10 (0.007)**
Blood chemistry								
TG	1.67 (1.23, 2.36)	1.20 (0.90, 1.83)	1.21 (0.78, 1.77)	1.83 (1.35, 2.66)	1.68 (1.25, 2.37)	22.619	**<0.001**	0.07 (0.08)
LDL-C	3.18 (2.72, 3.72)	3.12 (2.63, 3.79)	2.93 (2.67, 3.35)	3.20 (2.72, 3.64)	3.18 (2.72, 3.73)	1.579	0.664	0.03 (0.42)
FFA	520 (387.60, 678.10)	473.60 (389.80, 632)	485.35 (296.85, 649.30)	556 (434.60, 727.70)	515.10 (380.30, 676.50)	8.394	**0.039**	-0.02 (0.52)
ALT	33 (21, 58)	16 (11, 45)	23.50 (16, 36)	35 (24, 62)	33 (22, 58)	21.833	**<0.001**	**0.10 (0.008)**
Globulin	30.98 (26.82, 35.14)	30.16 (25.87, 34.45)	30.53 (26.35, 34.71)	30.58 (27.18, 33.98)	31.14 (26.86, 35.42)	0.890	0.446	**0.24 (<0.001)**
Uric Acid	409.85 (344, 485.10)	366.40 (305.30, 412.90)	350.75 (312.10, 479.10)	406.80 (334.70, 498.30)	416.50 (348.80, 487.80)	12.571	**0.006**	**0.10 (0.010)**
Cortisone at 8AM	12.87 (9.37, 17.09)	13.50 (8.41, 17.20)	12.37 (7.10, 16.72)	13.01 (9.43, 18.20)	12.75 (9.43, 17.03)	1.288	0.732	0.01 (0.79)
ACTH at 8 AM	32.82 (21.66, 47.53)	27.28 (17.16, 48.31)	32.24 (18.49, 46.76)	34.92 (22.26, 50.53)	32.73 (21.92, 46)	3.164	0.367	-0.00 (0.95)

BMI, body mass index. LDL-C, low-density lipoprotein cholesterol. TG, Triglycerides. FFA, Free Fatty Acid. ALT, Alanine Aminotransferase. ACTH, Adrenal Corticotropic Hormone. NAFLD, Non-alcoholic Fatty Liver Disease. NAFPD, Non-alcoholic Fatty Pancreas Disease. The categorical variable was compared using the Chi-squared test. Continuous variables were expressed as median (the first quantile, the third quantile) and were compared by the Kruskal–Wallis test, and their correlation with the class was assessed by Spearman correlation. All results in bold in the table indicate statistical significance.

### Patients with fatty liver exhibited higher fasting blood glucose, fasting insulin, and fasting C-peptide levels, and more pronounced insulin resistance

**Figure 2:**
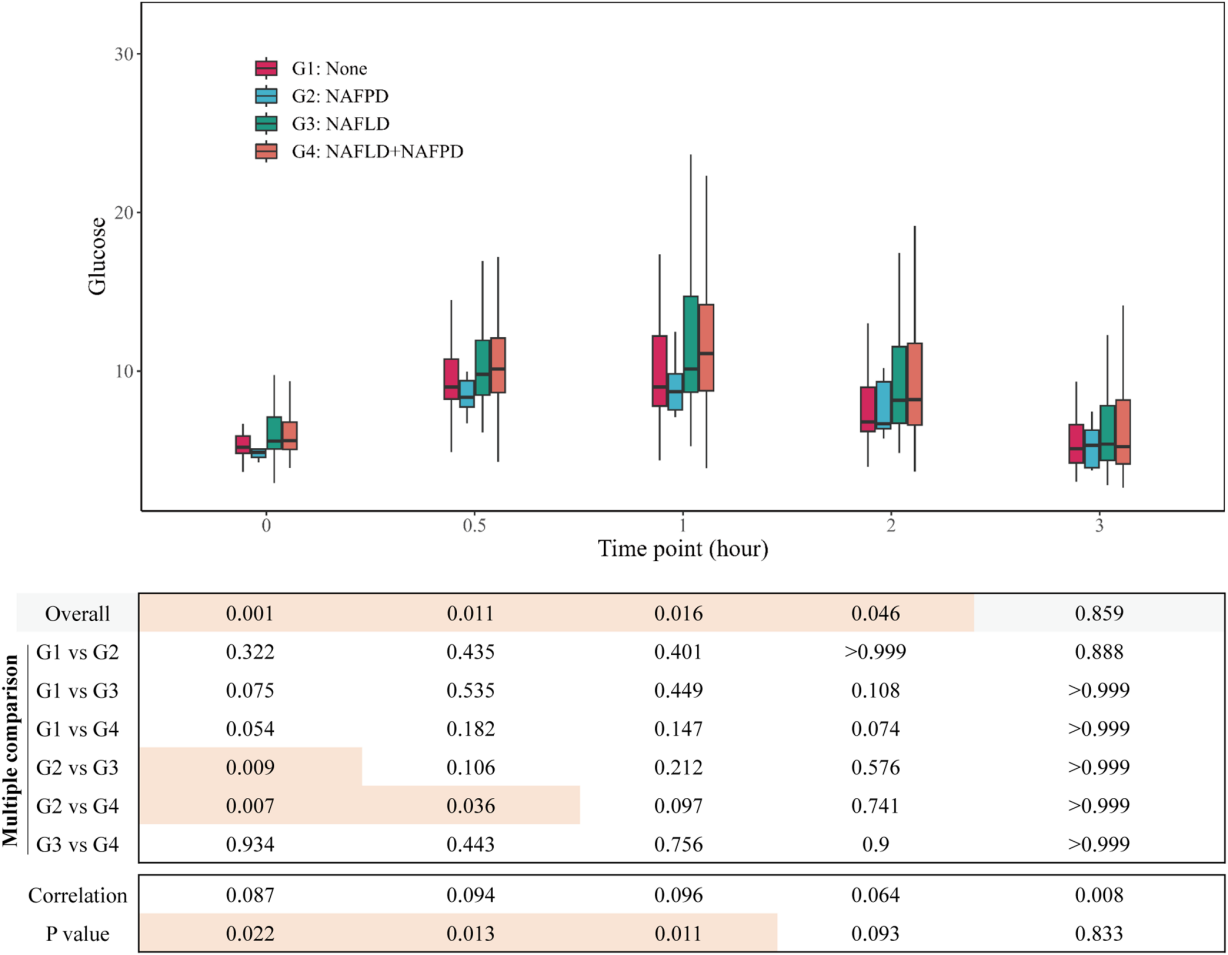
Group comparison of glucose at different time points.

**Figure 3:**
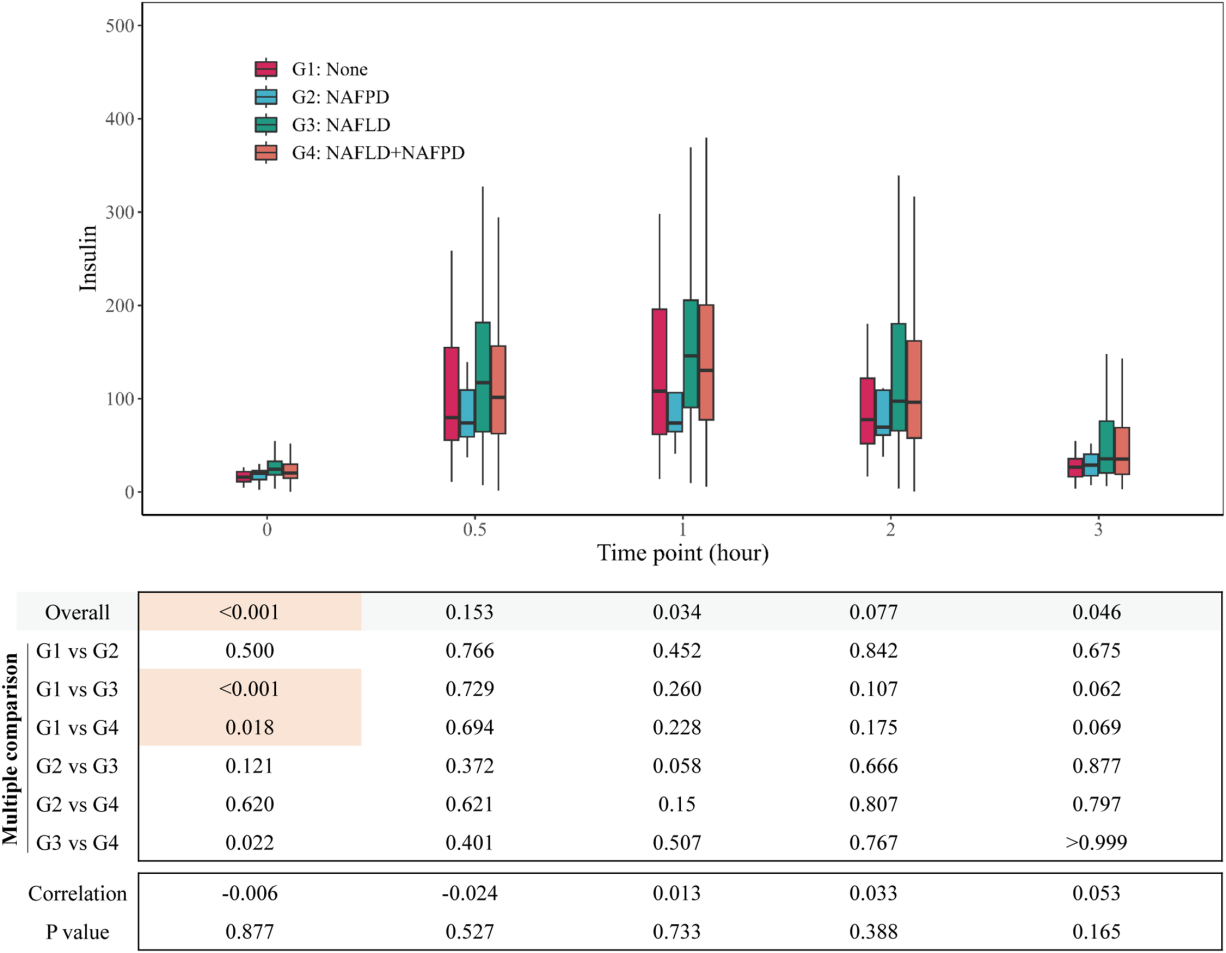
Group comparison of insulin at different time points.

**Figure 4:**
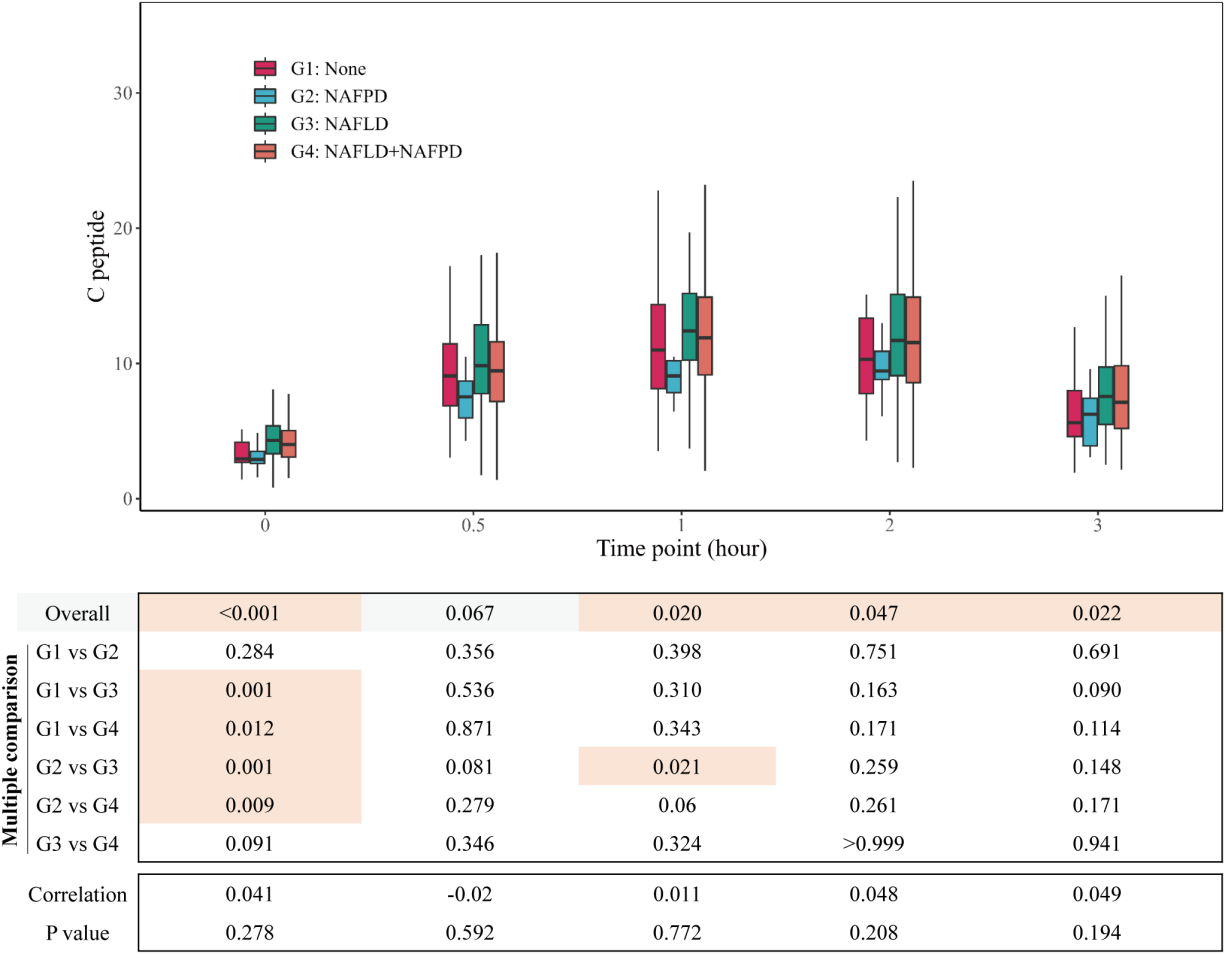
Group comparison of C-peptide at different time points.

The variations in glucose, insulin, and C-peptide profiles observed during the Oral OGTT were compared across the four groups. Significant differences were found in glucose levels at 0h, 0.5h, 1h, and 2h; insulin levels at 0h and 1h; and C-peptide levels at 0h, 1h, 2h, and 3h. Generally, patients in Group 3 and Group 4 exhibited higher levels of glucose, insulin, and C-peptide compared to Group 1 and Group 2 (**Table 2**). Further detailed comparisons between groups revealed more prominent differences primarily in the fasting state. Patients in Group 3 and Group 4 displayed elevated levels of fasting glucose (**Figure 2**), fasting insulin (**Figure 3**), and fasting C-peptide (**Figure 4**), along with increased values of the HOMA-IR, indicating heightened insulin resistance (**Table 2**, **Supplementary Figure S2**). Despite these variances, assessments of β-cell functionality, as measured by the Homeostatic Model Assessment of β-cell Function (HOMA-B) and the Oral Disposition Index (DIO), did not show significant differences among the four groups (**Table 2**, **Supplementary Figure S2**). The visceral fat area was higher in Group 3 and Group 4 (**Table 2**). These findings support the significant impact of concurrent fatty liver on glucose regulation, insulin levels, and insulin resistance in individuals with obesity. The correlational data highlights the specific role of fatty liver in influencing the glycemic and insulinemic profiles of subjects with obesity, thereby exacerbating insulin resistance.

**Table 2:** Outcomes of study population and group comparisons.

Characteristics	Overall (N=706)	None (n=39)	NAFPD (n=16)	NAFLD (n=110)	NAFLD+NAFPD (n=541)	Z/χ^2^	*P*	r (*P*)
MLFF	16.71 (11.25, 24.50)	3.13 (2.50, 4)	3.94 (2.88, 4.38)	15.69 (10.13, 25.88)	18.38 (13.36, 25.25)	157.578	**<0.001**	**0.36 (<0.001)**
S1	14 (8, 22)	3 (2, 4)	3.50 (2.50, 5)	14 (8, 23)	15 (9.40, 22)	140.117	**<0.001**	**0.30 (<0.001)**
S2	14 (8, 23)	3 (2, 4)	4 (3, 4)	15 (8, 24)	15.80 (10, 23)	142.339	**<0.001**	**0.32 (<0.001)**
S3	15 (9, 23)	3 (2, 4)	4 (3, 5.50)	15.50 (9, 24)	16 (10, 24)	143.288	**<0.001**	**0.31 (<0.001)**
S4	15 (10, 23)	3 (2, 4)	4.50 (3, 5)	14.50 (8, 24)	16.50 (12, 23.20)	148.896	**<0.001**	**0.33 (<0.001)**
S5	19 (12, 27)	3 (1.60, 4)	3 (2, 5)	17 (10, 24)	21 (14.70, 28)	162.048	**<0.001**	**0.40 (<0.001)**
S6	20 (12, 29)	3 (2, 4)	3 (2.50, 4)	16.50 (11, 26)	21 (16, 30)	165.012	**<0.001**	**0.41 (<0.001)**
S7	19 (13, 26)	4 (2.60, 5)	3.50 (3, 4.50)	17 (12, 27)	20 (14.20, 27)	152.187	**<0.001**	**0.35 (<0.001)**
S8	18 (12, 25.30)	3.60 (3, 4.40)	4 (2, 4)	18 (11, 26)	19.80 (14, 27)	152.768	**<0.001**	**0.35 (<0.001)**
Glucose								
0h	5.55 (5.04, 6.76)	5.20 (4.80, 6.21)	4.88 (4.56, 5.10)	5.59 (5.09, 7.10)	5.62 (5.06, 6.78)	16.315	**<0.001**	**0.09 (0.02)**
0.5h	9.93 (8.55, 12.02)	8.99 (8.21, 10.77)	8.35 (7.61, 9.48)	9.80 (8.49, 11.94)	10.13 (8.65, 12.10)	11.238	**0.011**	**0.09 (0.01)**
1h	10.74 (8.57, 14.02)	9 (7.80, 12.62)	8.70 (7.49, 9.84)	10.13 (8.67, 14.71)	11.10 (8.73, 14.19)	10.348	**0.016**	**0.10 (0.01)**
2h	8.01 (6.56, 11.48)	6.80 (6.13, 9.40)	6.68 (6.31, 9.51)	8.16 (6.71, 11.54)	8.20 (6.59, 11.81)	7.977	**0.046**	0.06 (0.09)
3h	5.26 (4.17, 8.06)	5.11 (4.04, 6.64)	5.33 (3.90, 6.52)	5.40 (4.37, 7.81)	5.24 (4.15, 8.22)	0.761	0.859	0.008 (0.83)
Insulin								
0h	20.65 (14.90, 30)	16 (11.10, 22.50)	20 (12, 24.80)	24.30 (18.30, 33)	20.40 (14.85, 29.85)	19.322	**<0.001**	-0.01 (0.88)
0.5h	103.65 (62.70, 157.65)	79.70 (54.50, 155.40)	74.10 (54.90, 123.10)	117.15 (64.40, 182.20)	101.40 (62.35, 156.45)	5.266	0.153	-0.02 (0.53)
1h	127.85 (77.80, 200.65)	108.20 (56.10, 209.40)	74 (63.90, 107.20)	145.85 (90, 205.70)	130.45 (77.35, 200.95)	8.678	**0.034**	0.01 (0.73)
2h	94.60 (58.60, 158.80)	77.50 (49.60, 121.90)	69.50 (60.90, 109.50)	97.45 (65.60, 182.40)	96 (57.70, 163.30)	6.840	0.077	0.03 (0.39)
3h	34.55 (18.85, 68.45)	26.50 (16.10, 37.60)	28.80 (12, 40.90)	35.60 (20.40, 76.40)	35.30 (18.95, 69.15)	8.008	0.046	0.05 (0.16)
C peptide								
0h	3.99 (2.98, 5.05)	2.95 (2.68, 4.25)	2.91 (2.58, 3.53)	4.31 (3.31, 5.39)	4.01 (3.08, 5.03)	23.381	**<0.001**	0.04 (0.28)
0.5h	9.46 (7.19, 11.80)	9.08 (6.56, 11.50)	7.53 (5.50, 9.21)	9.84 (7.74, 12.90)	9.46 (7.18, 11.60)	7.156	0.067	-0.02 (0.59)
1h	11.90 (9.08, 14.90)	11 (7.90, 15.40)	9.08 (7.67, 10.30)	12.40 (10.20, 15.20)	11.90 (9.15, 14.90)	9.839	**0.020**	0.01 (0.80)
2h	11.30 (8.68, 14.70)	10.30 (7.77, 13.70)	9.44 (8.57, 10.90)	11.70 (9.08, 15.10)	11.55 (8.56, 14.90)	7.953	**0.047**	0.05 (0.21)
3h	6.98 (5.16, 9.72)	5.62 (4.54, 8.25)	6.25 (3.81, 7.48)	7.56 (5.49, 9.74)	7.13 (5.19, 9.83)	9.635	**0.022**	0.05 (0.19)
HOMAIR	5.60 (3.74, 8.82)	3.65 (2.66, 5.72)	4.37 (2.97, 5.25)	6.86 (4.46, 10.02)	5.60 (3.79, 8.50)	20.720	**<0.001**	0.02 (0.56)
HOMA-β	199.04 (113.62, 305.78)	167.44 (112.00, 328.12)	266.24 (84.51, 373.83)	218.01 (129.15, 334.62)	194.51 (112.52, 299.63)	2.853	0.415	-0.04 (0.27)
DIO	0.94 (0.39, 1.74)	1.24 (0.57, 2.60)	1.15 (0.82, 1.92)	0.95 (0.39, 1.67)	0.89 (0.38, 1.70)	5.315	0.150	-0.06 (0.10)
MPFF	9.98 (6, 14.77)	3.27 (2, 3.67)	7.33 (6.33, 8.67)	3.67 (2.67, 4.33)	12 (8.67, 17)	361.195	**<0.001**	**0.69 (<0.001)**
VAT	168.89 (125.99, 222.33)	125.92 (91, 160.56)	114.15 (100.56, 152.42)	154.40 (120.58, 195.36)	173 (133.50, 231.81)	32.102	**<0.001**	**0.20 (<0.001)**

MLFF, Mean Liver Fat Fraction. HOMA-IR, Homeostatic model assessment for insulin resistance. HOMA-β, Homeostasis model assessment of beta cell function. DIO, Oral disposition index. NAFLD, Non-alcoholic Fatty Liver Disease. NAFPD, Non-alcoholic Fatty Pancreas Disease. VFA, Visceral Fat Area, Mean Pancreatic Fat Fraction, MPFF. The categorical variable was compared using the Chi-squared test. Continuous variables were expressed as median (the first quantile, the third quantile) and were compared by the Kruskal–Wallis test, and its correlation with the class was assessed by Spearman correlation. All results in bold in the table indicate statistical significance.

To investigate the impact of fatty pancreas on metabolic parameters in individuals with obesity, we analyzed the relationship between pancreatic fat, liver fat, and biomarkers measured during OGTT. Our results revealed a strong correlation between liver fat content and glucose levels at all time points during OGTT, as well as with insulin and C-peptide levels except at the 0.5-hour mark (**Supplementary Table S2**). In contrast, pancreatic fat content showed a weak association with fasting C-peptide levels, but no significant links to glucose levels or insulin and C-peptide levels at later stages of OGTT (**Supplementary Table S2**). Subgroup analysis of participants with obesity categorized by severity of fatty liver disease and extent of pancreatic fat infiltration indicated that in patients with mild fatty liver, pancreatic fat severity minimally affected fasting blood glucose (**Supplementary Figure S3**), with no impact on insulin and C-peptide levels at any time point (**Supplementary Figure S4, S5**). However, in individuals with moderate and severe fatty liver, pancreatic fat severity had little influence on glucose, insulin, and C-peptide levels throughout the OGTT (**Supplementary Figure S6-S11**).

### Indicators of higher glucose, insulin, and C peptide levels at 0h in patients with obesity

Multivariable regression analysis was performed to assess potential risk factors associated with elevated glucose, insulin, and C-peptide levels at 0h in individuals with obesity. The results highlighted that being female, older, having a higher BMI, elevated TG, FFA, and ALT levels, as well as lower LDL-C levels were all identified as risk factors for glucose levels at 0h. Additionally, NAFLD, younger age, higher BMI, and increased ALT levels were determined to be risk factors for insulin levels at 0h. Similarly, NAFLD, higher BMI, increased ALT and uric acid levels, and decreased LDL-C levels were found to be risk factors for C-peptide levels at 0h (**Table 3**). The study indicated that NAFLD and higher ALT levels were risk factors for fasting insulin and C-peptide, but not for fasting glucose, suggesting a potential direct influence of fatty liver on elevated insulin levels and an indirect effect on blood sugar through insulin and various metabolic markers. Importantly, multiple regression analysis confirmed that fatty pancreas was not a risk factor for fasting blood glucose, insulin, and C-peptide.

**Table 3:** Multivariable regression of glucose and insulin at 0 h on characteristics.

Characteristics	Glucose at 0h		Insulin at 0h		C peptide at 0h	
	Beta (95% CI)	*P*	Beta (95% CI)	*P*	Beta (95% CI)	*P*
Classification						
NAFPD + NAFLD	0.285 (-0.008, 0.579)	0.06	2.646 (-0.505, 5.798)	0.1	0.219 (-0.193, 0.632)	0.3
NAFLD	0.249 (-0.078, 0.576)	0.14	**6.733 (3.230, 10.237)**	**<0.001**	**0.613 (0.154, 1.071)**	**0.009**
NAFPD	-0.259 (-0.770, 0.252)	0.32	0.556 (-5.058, 6.170)	0.85	-0.652 (-1.386, 0.083)	0.08
None	ref.		ref.		ref.	
Sex						
Female	**0.192 (0.005, 0.379)**	**0.04**	-0.164 (-2.186, 1.858)	0.87	-0.135 (-0.400, 0.129)	0.32
Male	**ref.**		ref.		ref.	
Age	**0.025 (0.016, 0.034)**	**<0.001**	**-0.210 (-0.307, -0.112)**	**<0.001**	-0.005 (-0.018, 0.007)	0.41
BMI	**0.024 (0.011, 0.037)**	**<0.001**	**0.646 (0.504, 0.788)**	**<0.001**	**0.091 (0.072, 0.110)**	**<0.001**
Cortisone at 8h	0.010 (-0.005, 0.024)	0.19	-0.071 (-0.225, 0.083)	0.37	-0.000 (-0.020, 0.020)	0.98
ACTH at 8h	-0.001 (-0.005, 0.003)	0.59	0.013 (-0.028, 0.055)	0.53	-0.000 (-0.006, 0.005)	0.96
TG	**0.314 (0.175, 0.453)**	**<0.001**	0.054 (-1.436, 1.544)	0.94	0.162 (-0.033, 0.357)	0.1
LDL-C	**-0.293 (-0.449, -0.138)**	**<0.001**	-0.419 (-2.092, 1.253)	0.62	**-0.259 (-0.478, -0.040)**	**0.02**
FFA	**0.001 (0.001, 0.001)**	**<0.001**	-0.003 (-0.006, 0.001)	0.10	-0.000 (-0.001, 0.000)	0.16
ALT	**0.005 (0.003, 0.006)**	**<0.001**	**0.035 (0.017, 0.053)**	**<0.001**	**0.008 (0.006, 0.011)**	**<0.001**
Globulin	-0.004 (-0.008, 0.000)	0.07	0.026 (-0.016, 0.069)	0.23	-0.000 (-0.006, 0.005)	0.94
Uric acid	-0.000 (-0.001, 0.001)	0.66	0.002 (-0.005, 0.009)	0.6	**0.002 (0.001, 0.003)**	**<0.001**

CI, confidence interval. BMI, body mass index. LDL-C, low-density lipoprotein cholesterol. TG, Triglycerides. FFA, Free Fatty Acid. ALT, Alanine Aminotransferase. ACTH, Adrenal Corticotropic Hormone. NAFLD, Non-alcoholic Fatty Liver Disease. NAFPD, Non-alcoholic Fatty Pancreas Disease. The maximal variance inflation factor among included predictors was 4.01, which is less than 10. All results in bold in the table indicate statistical significance.

### Indicators of higher liver fat in patients with obesity

The study investigated the risk factors of fatty liver in patients with obesity and found that being female, younger, having higher C peptide at 0h, elevated ALT levels, increased pancreas fat, and higher visceral fat were all associated with higher liver fat levels (**Table 4**). The analysis revealed that while pancreatic fat may not directly impact blood sugar and insulin levels, it does influence liver fat accumulation. Additionally, the presence of visceral fat area also plays a role in affecting liver fat, suggesting a potential regulatory relationship between ectopic fats. These findings highlight the complex interplay of various fat deposits in metabolic changes among individuals with obesity.

**Table 4:** Multivariable regression of average liver fat on basic characteristics.

Characteristics	Average liver fat	
	Beta (95% CI)	P value
Sex		
**Female**	**2.568 (0.789, 4.347)**	**0.005**
Male	**ref.**	
Age	**-0.115 (-0.200, -0.030)**	**0.008**
BMI	-0.089 (-0.226, 0.048)	0.2
**C peptide at 0h**	**0.897 (0.443, 1.351)**	**<0.001**
Cortisone at 8h	-0.043 (-0.175, 0.088)	0.52
ACTH at 8h	-0.018 (-0.054, 0.017)	0.31
TG	-0.417 (-1.683, 0.850)	0.52
LDL-C	0.333 (-1.090, 1.755)	0.65
FFA	0.002 (-0.001, 0.005)	0.12
**ALT**	**0.068 (0.052, 0.084)**	**<0.001**
Globulin	0.008 (-0.029, 0.044)	0.68
Uric acid	0.003 (-0.003, 0.010)	0.3
**MPFF**	**0.429 (0.357, 0.500)**	**<0.001**
**VFA**	**0.011 (0.003, 0.019)**	**0.005**

CI, confidence interval. BMI, body mass index. LDL-C, low-density lipoprotein cholesterol. TG, Triglycerides. FFA, Free Fatty Acid. ALT, Alanine, VFA, Visceral Fat Area, Mean Pancreatic Fat Fraction, MPFF. Aminotransferase. ACTH, Adrenal Corticotropic Hormone. NAFLD, Non-alcoholic Fatty Liver Disease. NAFPD, Non-alcoholic Fatty Pancreas Disease. VFA, Visceral Fat Area, Mean Pancreatic Fat Fraction, MPFF. The maximal variance inflation factor among included predictors was 4.07, which is less than 10. All results in bold in the table indicate statistical significance.

These conclusions illustrate that amidst cohorts with obesity, the ramifications of fatty liver, particularly on metabolic aberrations such as blood glucose, triglycerides, uric acid levels, and insulin resistance, are more acutely manifested than those related to fatty pancreas. This insight accentuates the need for a focused evaluation and management of fatty liver in individuals with obesity to mitigate its pivotal contribution to metabolic dysregulation.

## Discussion

The findings of this study shed light on the heterogeneity of visceral fat deposition in obesity and its varied impacts on liver fat and overall metabolic health. By categorizing individuals with obesity based on the presence of NAFLD and fatty pancreas (NAFPD), we have identified significant variations in fat deposition and its associated metabolic consequences. Pooled data from 151 studies involving 101,028 participants indicate that NAFLD is prevalent in 69.99% of overweight and 70.90% of individuals with obesity, while non-alcoholic steatohepatitis (NASH) affects 33.50% of overweight and 33.67% of individuals with obesity (18). In contrast, fatty pancreas, which has been less extensively studied than fatty liver, shows a prevalence of approximately 16-35% in the general population (19, 20). In our cohort, the prevalence of NAFLD was 92.21%, and NAFPD was observed in 78.89% of individuals with obesity. Notably, while 5.52% of the participants displayed neither condition, a substantial 76.62% were affected by both NAFLD and NAFPD.

The prevalence of both NAFLD and NAFPD within the majority of the study population highlights the common co-occurrence of these conditions in obesity. However, the study reveals that fatty liver has a more substantial impact on metabolic abnormalities compared to fatty pancreas. Groups with fatty liver exhibited severe insulin resistance, as evidenced by elevated fasting blood glucose, insulin, C-peptide levels, and higher HOMA-IR values. These findings align with existing research that underscores hepatic steatosis as a key factor in the development of insulin resistance and type 2 diabetes (9, 10, 14, 21). The liver’s central role in glucose and lipid metabolism means that its dysfunction due to fat accumulation can have extensive systemic effects (9, 22–24). This is particularly pertinent given the rising prevalence of NAFLD among populations with obesity and its potential progression to more severe liver diseases (25–27), highlighting the critical need for early detection and management to curb related metabolic complications.

In contrast, much is still unknown about the pathological mechanisms of NAFPD and its effects on blood sugar and insulin resistance (19, 28). While studies suggest that pancreatic fat is not causally linked to the risk of type 2 diabetes (10) and does not affect insulin secretion in people with normal glucose tolerance (11), it is noted that pancreatic fat levels are elevated in individuals with prediabetes and type 2 diabetes (10, 29), and there is an inverse relationship between pancreatic fat and insulin secretion capability in those with impaired fasting glucose or glucose tolerance (11) and in people with type 2 diabetes (30). Nevertheless, pancreatic fat has not shown a significant correlation with insulin resistance (14, 30). In this study, NAFPD has not shown a significant influence on glucose levels or insulin resistance.

Visceral fat accumulation heterogeneity is influenced by multiple factors including genetic predispositions, hormonal levels, and dietary components (31–33). Subcutaneous and visceral adipose tissues differ fundamentally in metabolic functions, with visceral fat more significantly impacting metabolic health due to its proximity to the liver and its higher inflammatory and lipolytic activity (31–33). Sex hormones and local cortisol production play critical roles in regional fat distribution, exacerbating visceral adiposity in response to caloric excess (31). This heterogeneity in fat deposition underscores the complex interplay between lifestyle, biological factors, and metabolic risk.

Interestingly, the measures of β cell function did not significantly differ across the groups in this study, which might suggest that the impact of ectopic fat on β cell function is less direct or occurs later in the disease progression. This observation warrants further investigation, as it may provide insights into the temporal relationship between ectopic fat deposition and β cell dysfunction.

The heterogeneity observed in this study also points to the potential for personalized medical approaches in treating obesity and its related complications. Understanding the specific patterns of fat deposition can guide more targeted interventions, potentially improving treatment outcomes (31–33). For instance, individuals with predominant fatty liver might benefit more from interventions focusing on reducing hepatic fat (34, 35), while those with predominant pancreatic fat might require different strategies. Recent advances have significantly improved treatments targeting hepatic and pancreatic fat reduction. Chih Hung Lo et al. demonstrated that nano-carriers with high selective targeting capabilities can effectively deliver therapeutic agents to fatty tissues, substantially reducing fat content in the liver and pancreas. These findings underscore the therapeutic potential of lysosome-acidifying tetrafluoro succinic acid (TFSA) nanoparticles for the treatment of T2DM (13). Moreover, the study by Zeng J et al. revealed new therapeutic potentials through the use of acid nanoparticles (acNPs), which inhibit impaired lysosomal acidification by restoring lysosomal acidity (36). Collectively, these findings highlight the promising clinical applications of nanotechnology and biotechnological approaches in reducing hepatic and pancreatic fat.

However, this study is not without limitations. A primary limitation is the uneven distribution of participants across the four groups based on fat deposition, with a particularly small sample size for the NAFPD group (n=16), representing only 2% of the total cohort. The small sample size for the NAFPD group may limit the statistical power and the generalizability of our findings related to this specific subgroup. This small sample size may compromise the representativeness of our findings for the NAFPD group and potentially introduces a sampling bias, as the results may not be generalizable to other populations with a different distribution of fat deposition. Consequently, the conclusions drawn from the NAFPD group should be interpreted with caution. We also recommend that future studies consider employing strategies to enhance the recruitment and retention of participants in underrepresented subgroups to ensure a more balanced sample distribution. The cross-sectional nature limits the ability to infer causality or the direction of the relationships observed. Longitudinal studies are needed to better understand the progression of these conditions and their long-term implications. Additionally, the study population may not be representative of all individuals with obesity, particularly considering the trends of more females in our research groups and the variations in obesity phenotypes across different ethnicities and age groups.

## Conclusion

In conclusion, this study contributes to a growing body of evidence highlighting the complexity of fat distribution in obesity and its differential impact on metabolic health. It emphasizes the need for a more nuanced approach to understanding and managing obesity, considering the individual variations in ectopic fat deposition and their metabolic implications.

## Data Availability

The original contributions presented in the study are included in the article/**Supplementary Material**. Further inquiries can be directed to the corresponding authors.
