# Corrigendum: Exploring the heterogeneity of hepatic and pancreatic fat deposition in obesity: implications for metabolic health

**DOI:** 10.3389/fendo.2024.1514364

**Published:** 2024-11-20

**Authors:** Ming Deng, Zhen Li, Shangyu Chen, Huawei Wang, Li Sun, Jun Tang, Liman Luo, Xiaoxiao Zhang, Haibo Xu, Zhe Dai

**Affiliations:** ^1^ Department of Radiology, Zhongnan Hospital of Wuhan University, Wuhan University, Wuhan, China; ^2^ Hubei Provincial Engineering Research Center of Multimodal Medical Imaging Technology and Clinical Application, Wuhan, China; ^3^ Wuhan Clinical Research and Development Center of Brain Resuscitation and Functional Imaging, Wuhan, China; ^4^ Department of Hepatobiliary and Pancreatic Surgery, Zhongnan Hospital of Wuhan University, Wuhan University, Wuhan, China; ^5^ Department of Endocrinology, Zhongnan Hospital of Wuhan University, Wuhan University, Wuhan, China; ^6^ Department of Endocrinology and Metabolism, Guangxi Academy of Medical Sciences and the People’s Hospital of Guangxi Zhuang Autonomous Region, Guangxi, Nanning, China; ^7^ Department of MSC Clinical & Technical Solutions, Philips Healthcare, Beijing, China; ^8^ Department of Clinical Nutrition, Zhongnan Hospital of Wuhan University, Wuhan University, Wuhan, China

**Keywords:** obesity, non-alcoholic fatty liver disease, non-alcoholic fatty pancreas disease, insulin resistance, fat deposition heterogeneity

In the published article, there was an error in the legend for **Figure 1** as published. In the original article, there was a misplacement of data in the text description for **Figure 1** as published. The corrected caption and its figure appear below.

**Figure 1:**
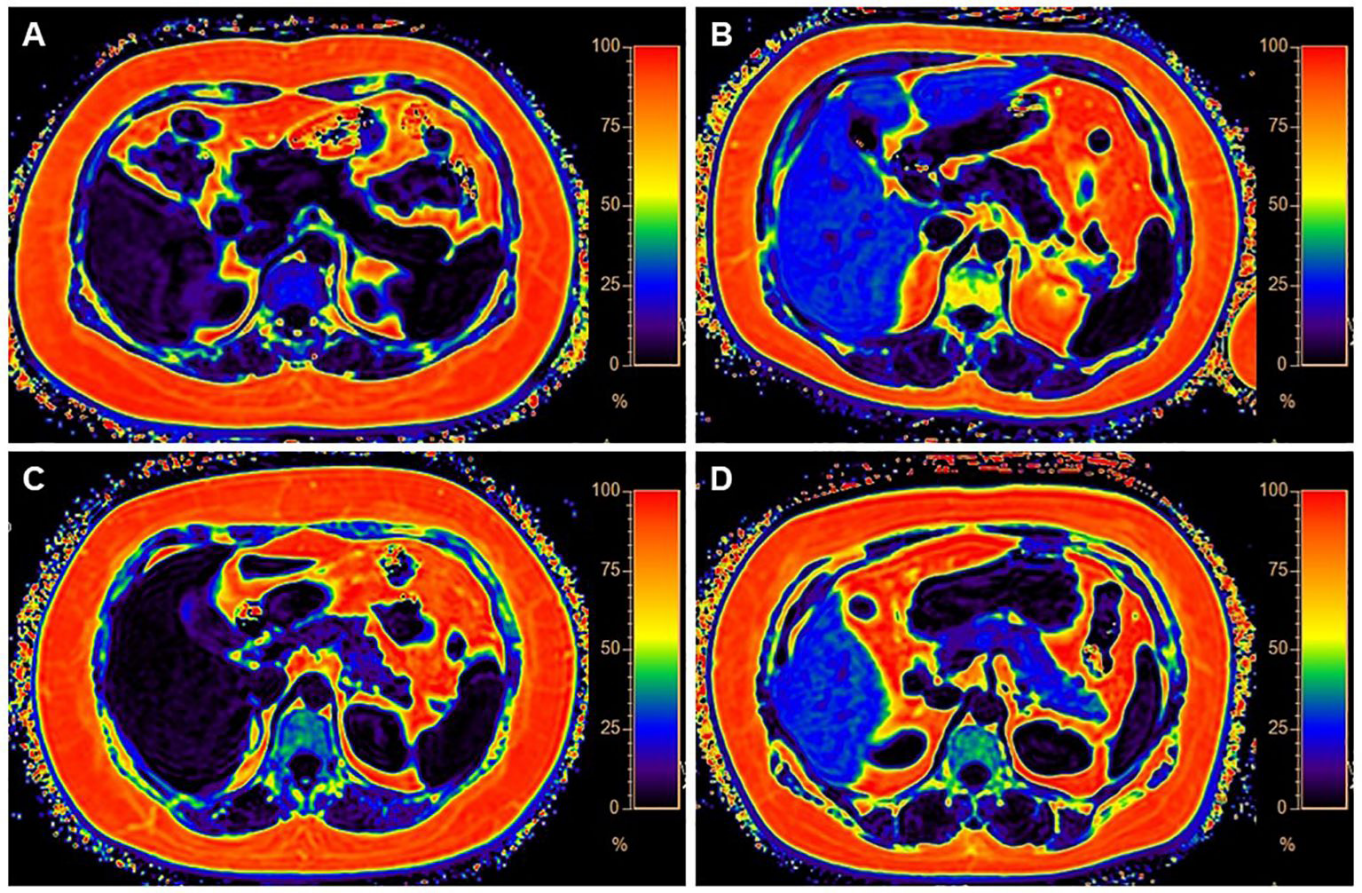
Four categories of MR images illustrate different levels of fat deposition in obesity. **(A)** Represents the first category, showing no fat deposition in either the liver or pancreas. The average fat fraction (FF) of the liver and pancreas were 4.37% and 1%, respectively. **(B)** Represents the second category, with high-fat deposition in the liver and minimal fat deposition in the pancreas. The average FF of the liver and pancreas were 28% and 4%, respectively. **(C)** Illustrates the third category, with less fat deposition in the liver but higher fat deposition in the pancreas. The average FF of the liver and pancreas were 3% and 13.1%, respectively. **(D)** Demonstrates the fourth category, characterized by fatty deposits in both the liver and pancreas. The average FF of the liver and pancreas were 31% and 22.6%, respectively.

The authors apologize for this error and state that this does not change the scientific conclusions of the article in any way. The original article has been updated.

