# Supplementary material for: Exploring the heterogeneity of hepatic and pancreatic fat deposition in obesity: implications for metabolic health
Source: Front Endocrinol (Lausanne). 2024 Oct 8;15:1447750. doi: 10.3389/fendo.2024.1447750 (PMC11493592; doi:10.3389/fendo.2024.1447750)
Supplement: Supplementary file 1 [file DataSheet1.docx]

**Supplement figures**


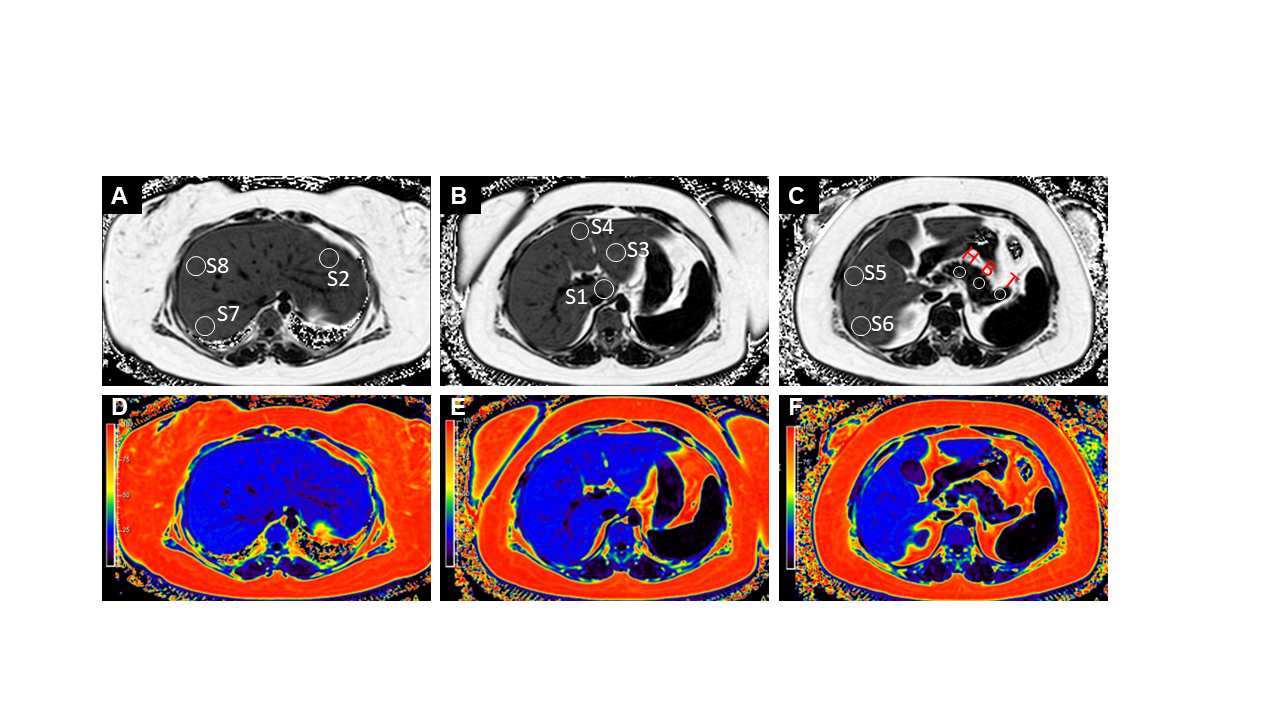


**Figure S1**. Quantitative measurement of liver and pancreatic fat and region of interest (ROI) selection methods. Anatomical localization of the liver and pancreas was conducted based on the fat fraction (A, B, and C) and colored (D, E, and F) maps generated from abdominal MRI. The liver was divided into eight segments, designated as S1-S8, and the corresponding ROIs were placed on S1-S8 while avoiding the hepatic veins and portal veins as much as possible. For the determination of pancreatic fat fraction, the pancreas was divided into the head, body, and tail, abbreviated as H/B/T. Subsequently, the corresponding ROIs were placed, and the average value of the ROIs in the H/B/T regions was used to calculate the pancreatic fat content.


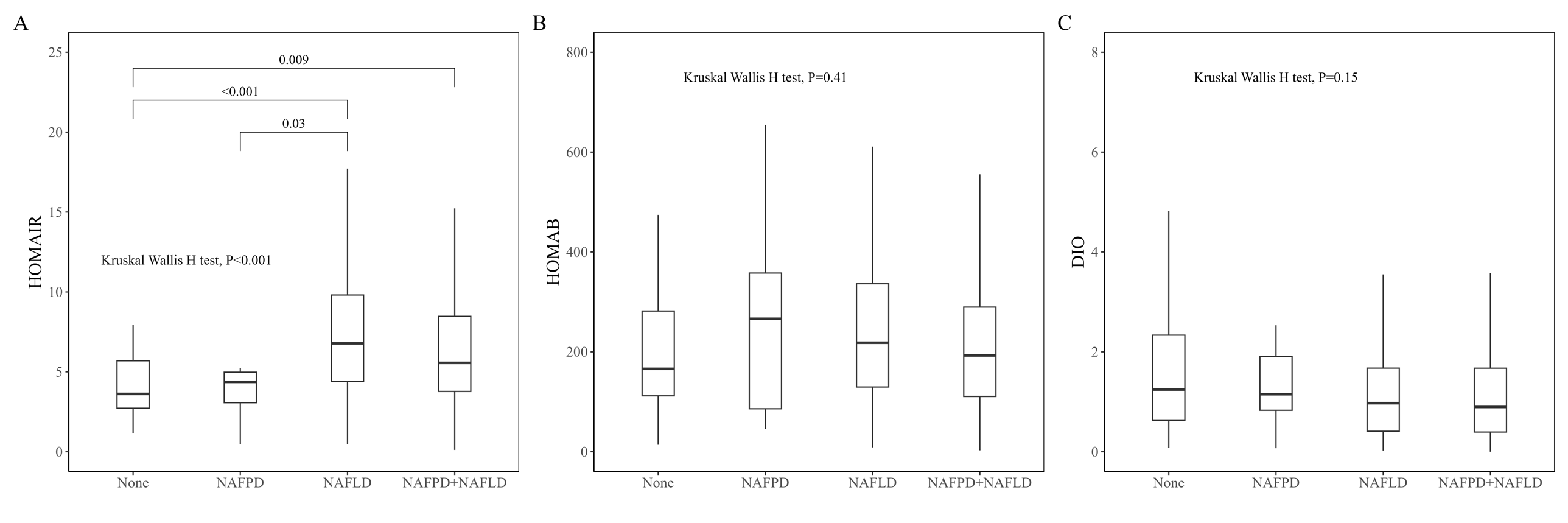


**Figure S2.** Comparative analysis of HOMAIR (A), HOMAB (B), and DIO (C) levels across four subgroups: none (no NAFLD and NAFPD), NAFPD (with NAFPD, no NAFLD), NAFLD (with NAFLD, no NAFPD), and NAFPD+NAFLD (with both NAFPD and NAFLD).


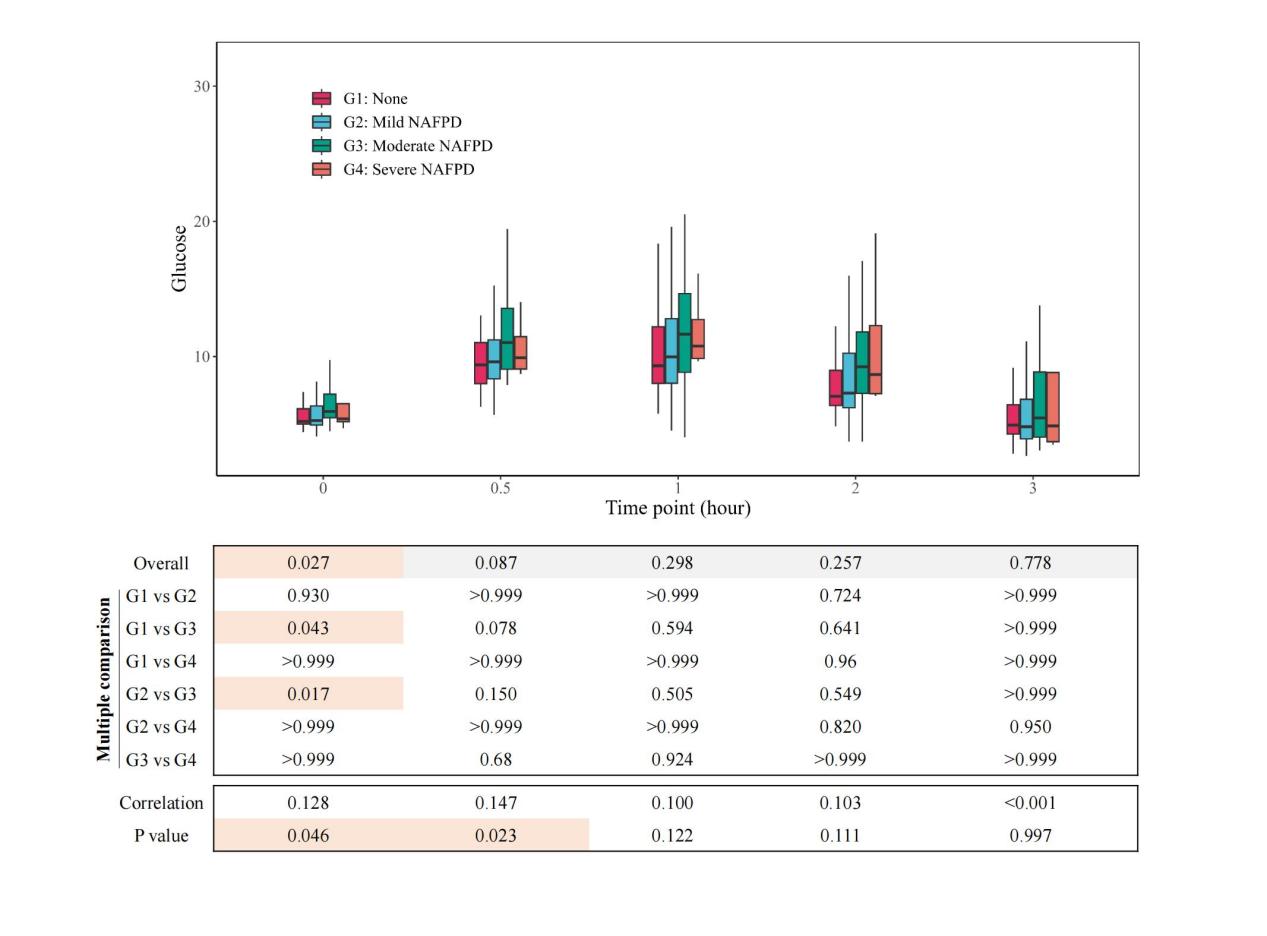


**Figure S3**. Comparative analysis of glucose levels during OGTT in subjects with mild NAFLD. This figure illustrates the glucose level variations across four subgroups: G1 (no NAFPD), G2 (mild NAFPD), G3 (moderate NAFPD), and G4 (severe NAFPD).


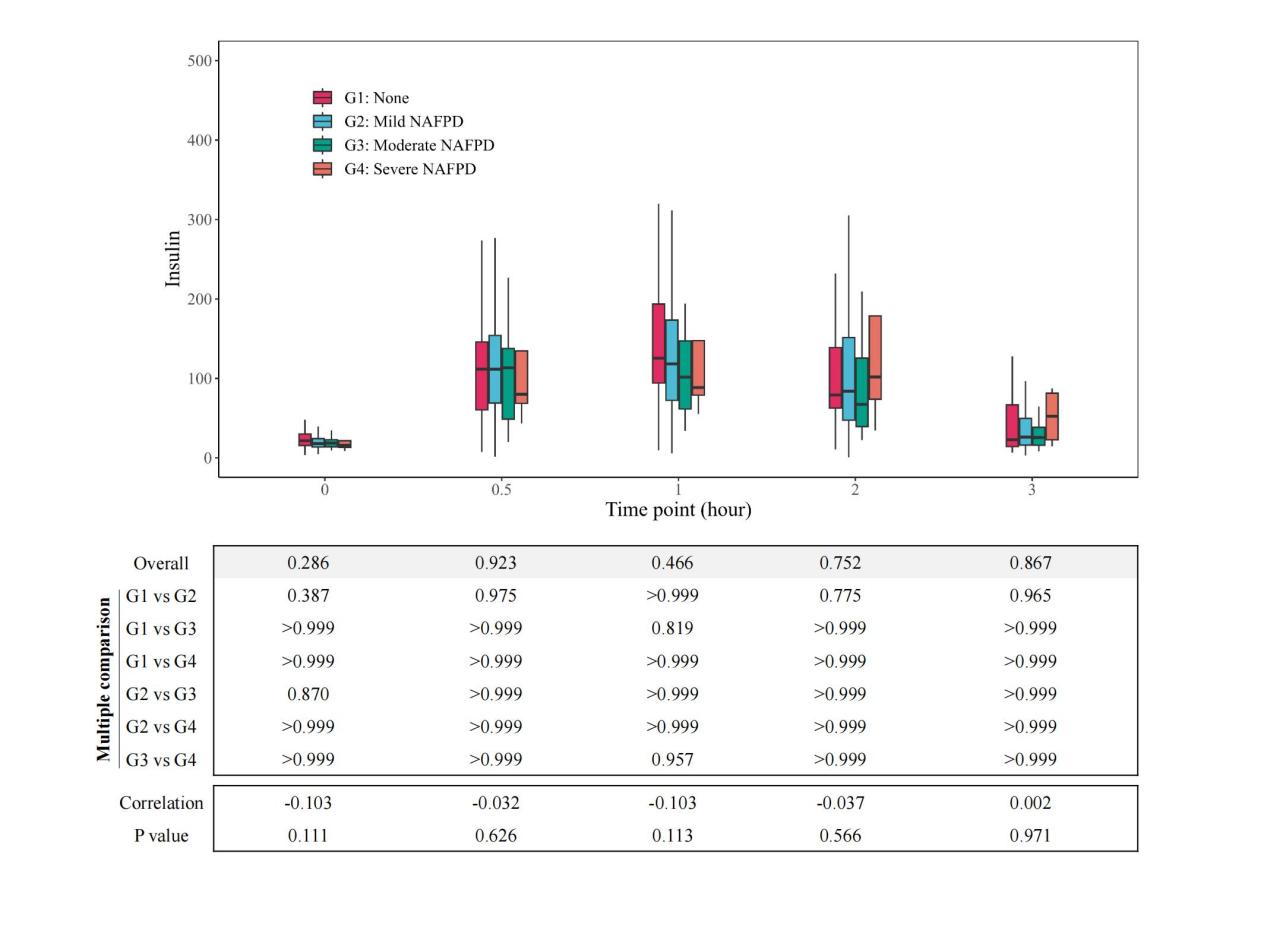


**Figure S4**. Comparative analysis of insulin levels during OGTT in subjects with mild NAFLD. This figure illustrates the insulin level variations across four subgroups: G1 (no NAFPD), G2 (mild NAFPD), G3 (moderate NAFPD), and G4 (severe NAFPD).


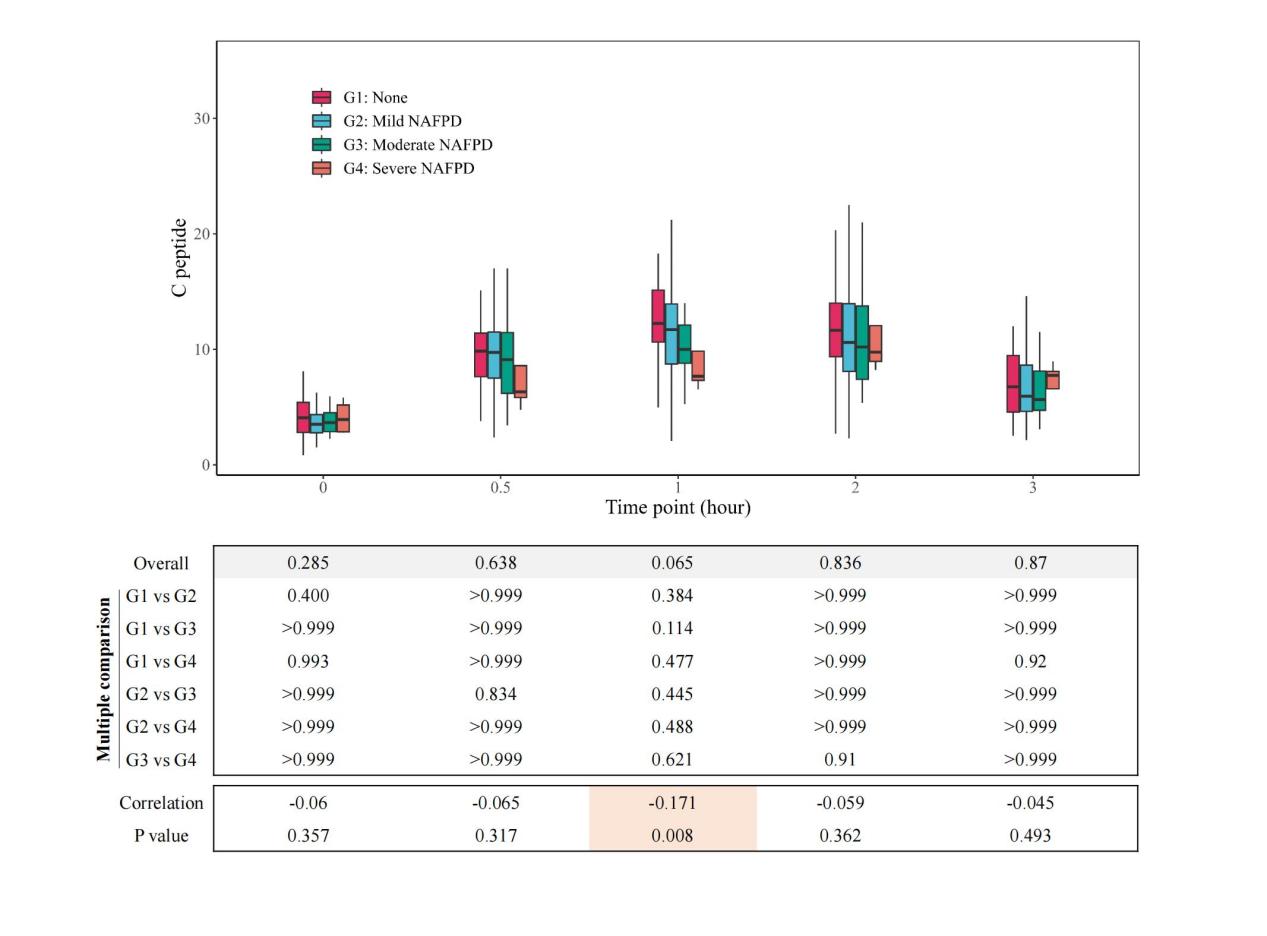


**Figure S5**. Comparative analysis of C peptide levels during OGTT in subjects with mild NAFLD. This figure illustrates the C peptide level variations across four subgroups: G1 (no NAFPD), G2 (mild NAFPD), G3 (moderate NAFPD), and G4 (severe NAFPD).


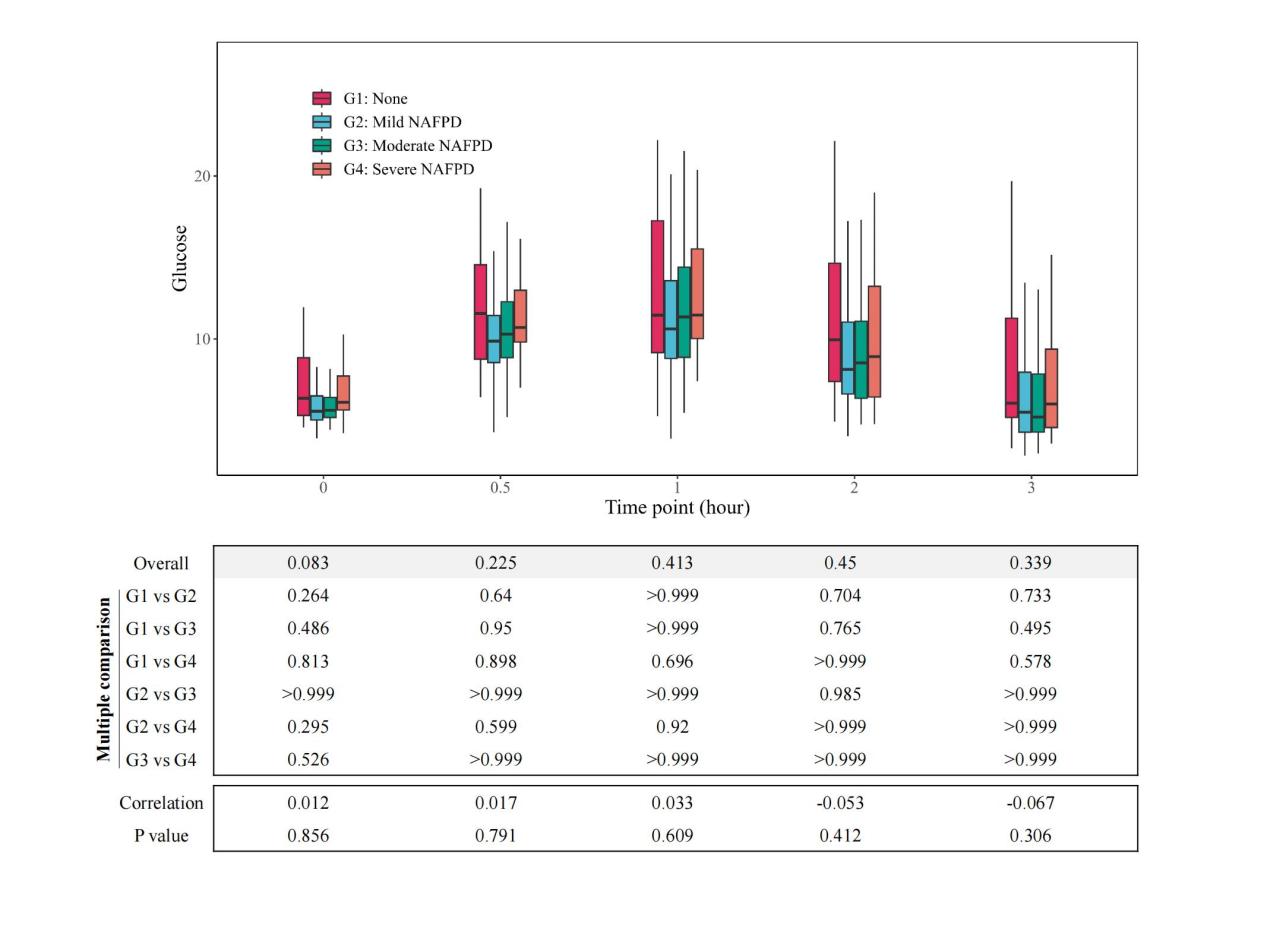


**Figure S6**. Comparative analysis of glucose levels during OGTT in subjects with moderate NAFLD. This figure illustrates the glucose level variations across four subgroups: G1 (no NAFPD), G2 (mild NAFPD), G3 (moderate NAFPD), and G4 (severe NAFPD).


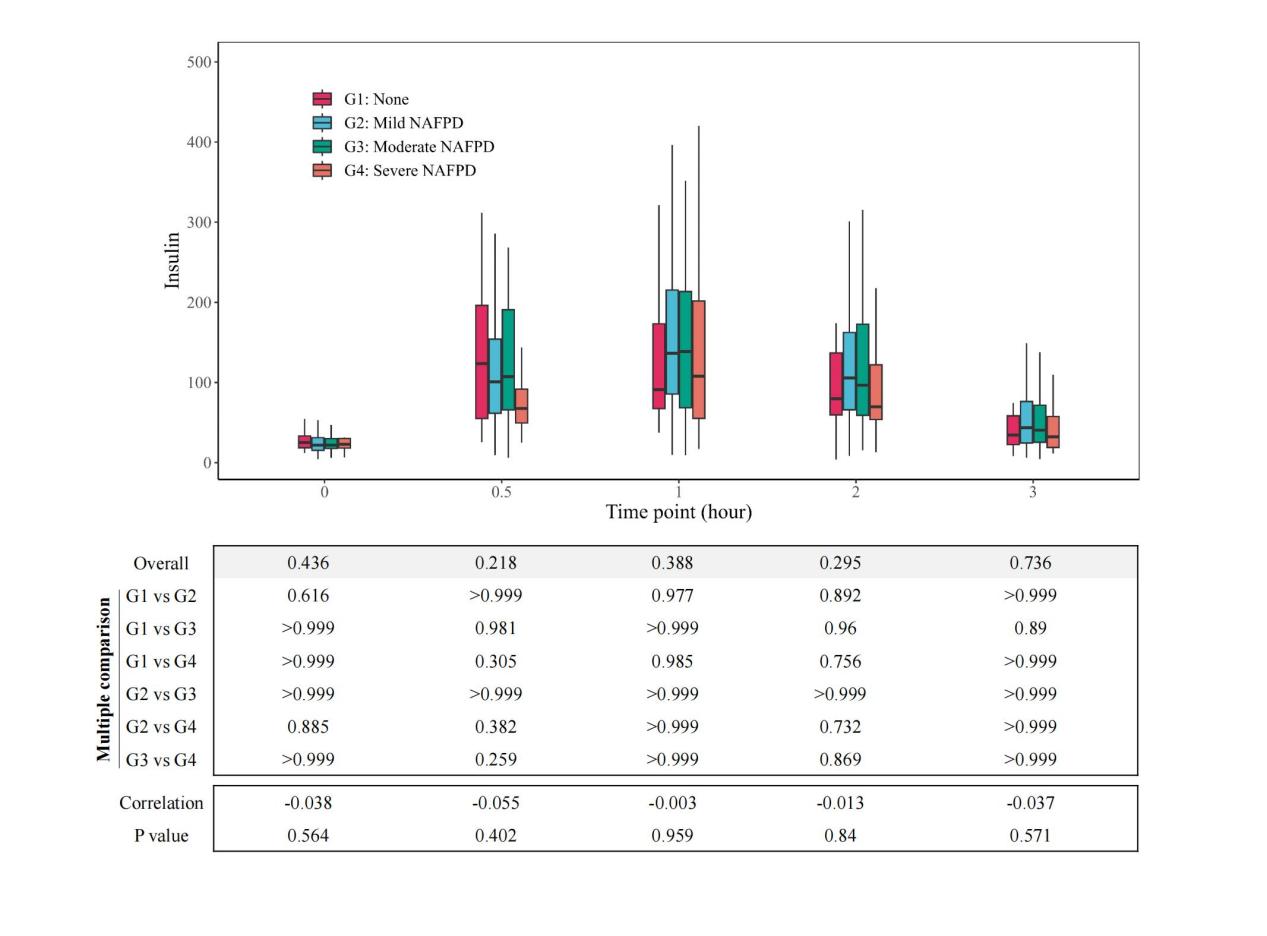


**Figure S7**. Comparative analysis of insulin Levels during OGTT in subjects with moderate NAFLD. This figure illustrates the insulin level variations across four subgroups: G1 (no NAFPD), G2 (mild NAFPD), G3 (moderate NAFPD), and G4 (severe NAFPD).


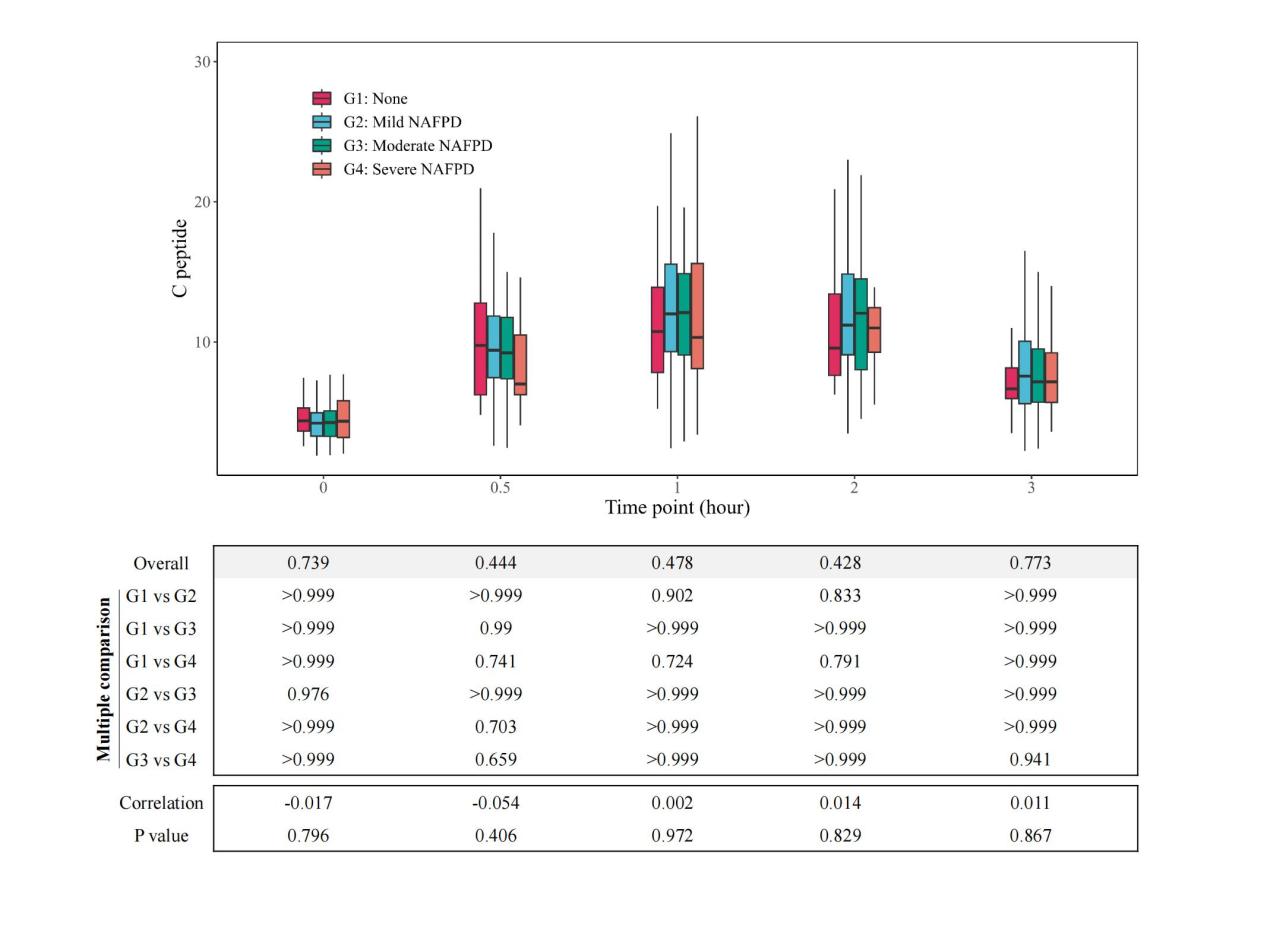


**Figure S8.** Comparative analysis of C peptide levels during OGTT in subjects with moderate NAFLD. This figure illustrates the C peptide level variations across four subgroups: G1 (no NAFPD), G2 (mild NAFPD), G3 (moderate NAFPD), and G4 (severe NAFPD).


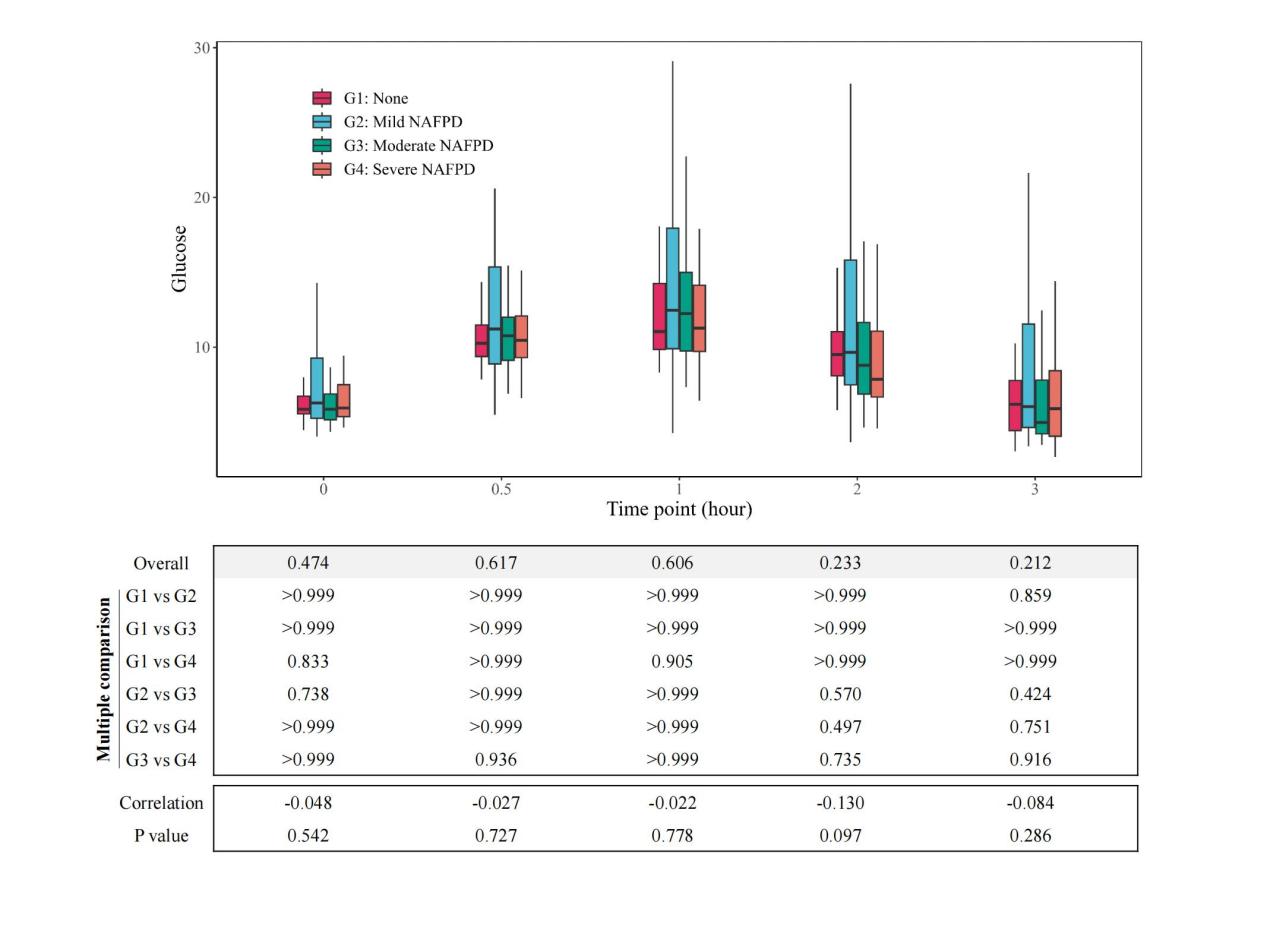


**Figure S9**. Comparative analysis of glucose levels during OGTT in subjects with severe NAFLD. This figure illustrates the glucose level variations across four subgroups: G1 (no NAFPD), G2 (mild NAFPD), G3 (moderate NAFPD), and G4 (severe NAFPD).


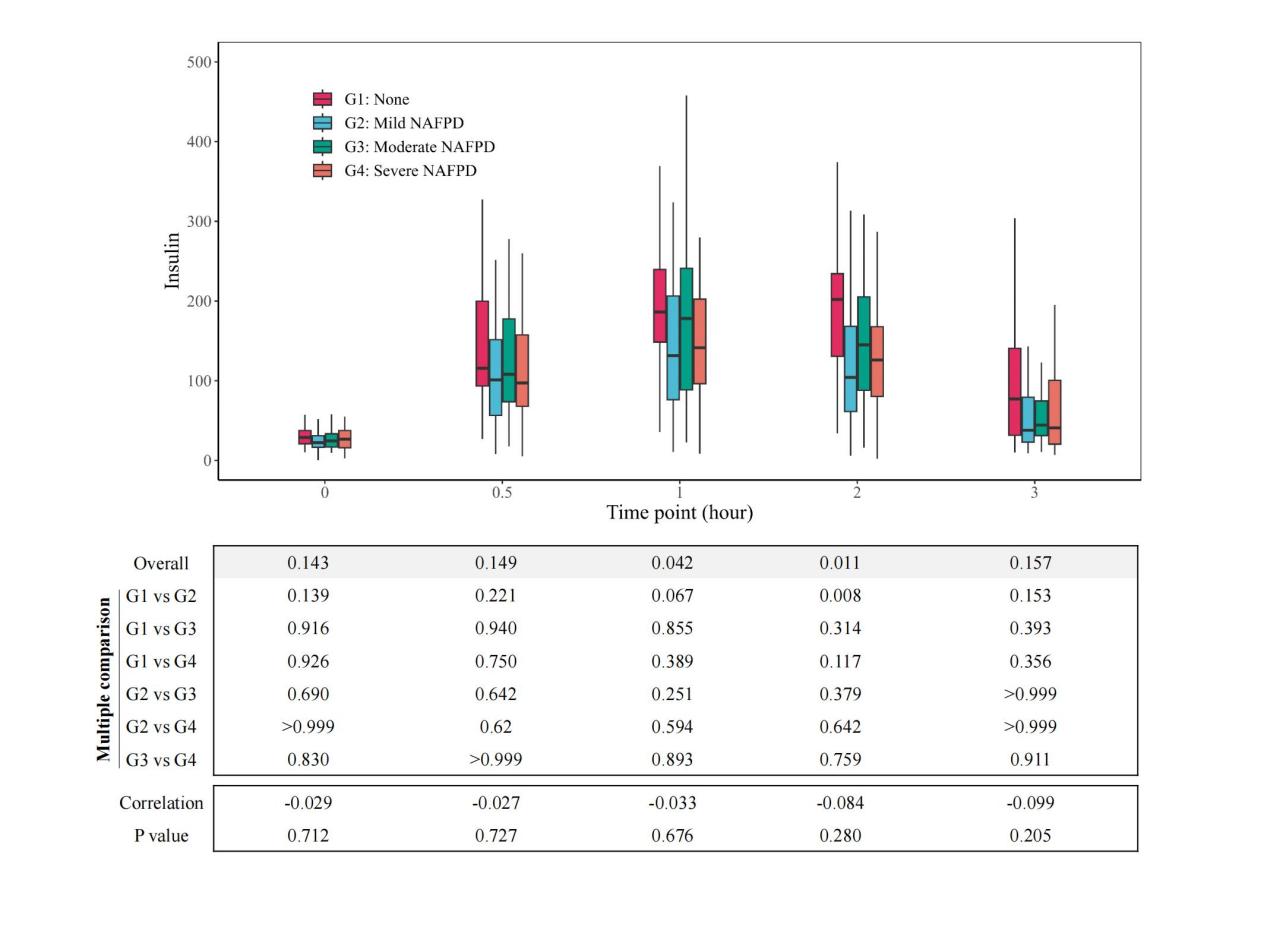


**Figure S10**. Comparative analysis of insulin levels during OGTT in subjects with severe NAFLD. This figure illustrates the insulin level variations across four subgroups: G1 (no NAFPD), G2 (mild NAFPD), G3 (moderate NAFPD), and G4 (severe NAFPD).


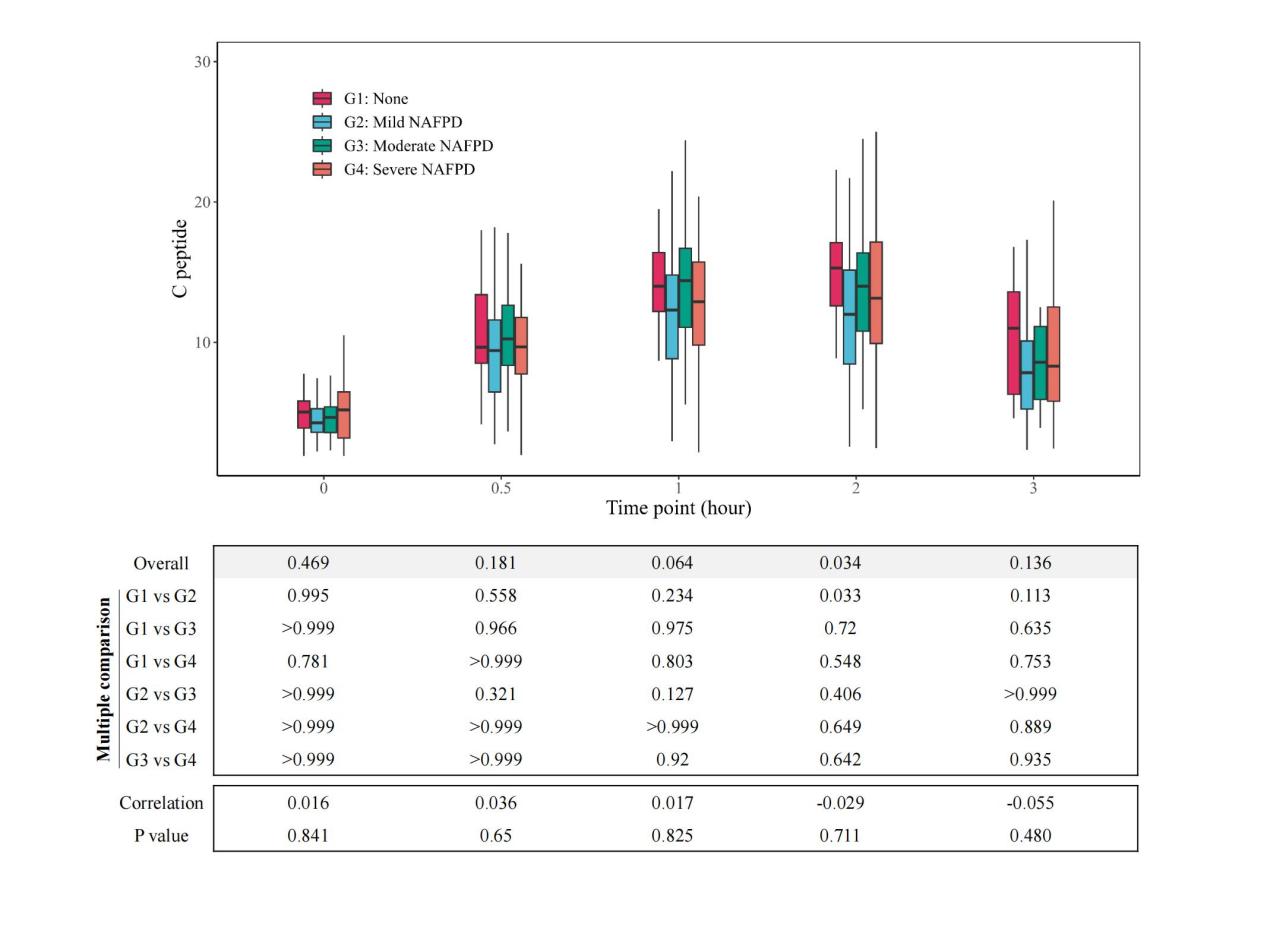


**Figure S11**. Comparative analysis of C peptide levels during OGTT in subjects with severe NAFLD. This figure illustrates the C peptide level variations across four subgroups: G1 (no NAFPD), G2 (mild NAFPD), G3 (moderate NAFPD), and G4 (severe NAFPD).
