# Supplementary material for: Exploring the heterogeneity of hepatic and pancreatic fat deposition in obesity: implications for metabolic health
Source: Front Endocrinol (Lausanne). 2024 Oct 8;15:1447750. doi: 10.3389/fendo.2024.1447750 (PMC11493592; doi:10.3389/fendo.2024.1447750)
Supplement: Supplementary file 2 [file Table1.docx]

**Table S1** Stratification of Participants Based on Hepatic and Pancreatic Fat Deposition

| **Group** | **Description** | **Criteria** |
| --- | --- | --- |
| G1 | None | No fat deposition |
| G2 | NAFPD | Fatty pancreas only |
| G3 | NAFLD | Fatty liver only |
| G4 | NAFLD+NAFPD | Both conditions |

**Note:** NAFLD, Non-alcoholic Fatty Liver Disease. NAFPD, Non-alcoholic Fatty Pancreas Disease.

**Table S2** Spearman correlation of liver fat and pancreas fat with glucose, insulin and C peptide during OGTT

| Variable | Time point | r (*P*) | |
| --- | --- | --- | --- |
|  |  | Average liver fat | Average pancreas fat |
| Glucose | 0h | 0.12(<0.001) | 0.01(0.883) |
|  | 0.5h | 0.13(<0.001) | 0.02(0.557) |
|  | 1h | 0.17(<0.001) | 0.03(0.389) |
|  | 2h | 0.14(<0.001) | -0.01(0.848) |
|  | 3h | 0.10(0.008) | -0.02(0.585) |
| Insulin | 0h | 0.19(<0.001) | 0.03(0.372) |
|  | 0.5h | 0.008(0.826) | -0.03(0.426) |
|  | 1h | 0.12(0.002) | 0.02(0.621) |
|  | 2h | 0.17(<0.001) | 0.02(0.655) |
|  | 3h | 0.21(<0.001) | 0.00(0.961) |
| C peptide | 0h | 0.27(<0.001) | 0.10(0.012) |
|  | 0.5h | 0.04(0.335) | -0.02(0.646) |
|  | 1h | 0.12(0.002) | 0.03(0.474) |
|  | 2h | 0.18(<0.001) | 0.05(0.220) |
|  | 3h | 0.25(<0.001) | 0.03(0.431) |
